# Genome-wide Association Study and Meta-Analysis Identify *ISL1* as Genome-wide Significant Susceptibility Gene for Bladder Exstrophy

**DOI:** 10.1371/journal.pgen.1005024

**Published:** 2015-03-12

**Authors:** Markus Draaken, Michael Knapp, Tracie Pennimpede, Johanna M. Schmidt, Anne-Karolin Ebert, Wolfgang Rösch, Raimund Stein, Boris Utsch, Karin Hirsch, Thomas M. Boemers, Elisabeth Mangold, Stefanie Heilmann, Kerstin U. Ludwig, Ekkehart Jenetzky, Nadine Zwink, Susanne Moebus, Bernhard G. Herrmann, Manuel Mattheisen, Markus M. Nöthen, Michael Ludwig, Heiko Reutter

**Affiliations:** 1 Institute of Human Genetics, University of Bonn, Bonn, Germany; 2 Department of Genomics, Life & Brain Center, University of Bonn, Bonn, Germany; 3 Institute of Medical Biometry, Informatics, and Epidemiology, University of Bonn, Bonn, Germany; 4 Department of Developmental Genetics, Max Planck Institute for Molecular Genetics, Berlin, Germany; 5 Department of Urology and Pediatric Urology, University Hospital of Ulm, Germany; 6 Department of Pediatric Urology, St. Hedwig Hospital Barmherzige Brüder, Regensburg, Germany; 7 Department of Urology, Division of Pediatric Urology, University of Mainz, Mainz, Germany; 8 Department of General Pediatrics and Neonatology, Justus Liebig University, Giessen, Germany; 9 Department of Urology, Division of Paediatric Urology, University of Erlangen-Nürnberg, Erlangen, Germany; 10 Department of Pediatric Surgery and Pediatric Urology, Children’s Hospital of Cologne, Cologne, Germany; 11 Department of Clinical Epidemiology and Aging Research, German Cancer Research Center, Heidelberg, Germany; 12 Department of Child and Adolescent Psychiatry and Psychotherapy, Johannes-Gutenberg University, Mainz, Germany; 13 Institute of Medical Informatics, Biometry, and Epidemiology, University Hospital of Essen, University Duisburg-Essen, Essen, Germany; 14 Department of Biostatistics, Harvard School of Public Health, Boston, Massachusetts, United States of America; 15 Department of Biomedicine, Aarhus University, Aarhus, Denmark; 16 Department of Genomic Mathematics, University of Bonn, Bonn, Germany; 17 Department of Clinical Chemistry and Clinical Pharmacology, University of Bonn, Bonn, Germany; 18 Department of Neonatology, Children's Hospital, University of Bonn, Bonn, Germany; Monash University, AUSTRALIA

## Abstract

The bladder exstrophy-epispadias complex (BEEC) represents the severe end of the uro-rectal malformation spectrum, and is thought to result from aberrant embryonic morphogenesis of the cloacal membrane and the urorectal septum. The most common form of BEEC is isolated classic bladder exstrophy (CBE). To identify susceptibility loci for CBE, we performed a genome-wide association study (GWAS) of 110 CBE patients and 1,177 controls of European origin. Here, an association was found with a region of approximately 220kb on chromosome 5q11.1. This region harbors the *ISL1* (*ISL LIM homeobox 1*) gene. Multiple markers in this region showed evidence for association with CBE, including 84 markers with genome-wide significance. We then performed a meta-analysis using data from a previous GWAS by our group of 98 CBE patients and 526 controls of European origin. This meta-analysis also implicated the 5q11.1 locus in CBE risk. A total of 138 markers at this locus reached genome-wide significance in the meta-analysis, and the most significant marker (rs9291768) achieved a P value of 2.13 × 10^−12^. No other locus in the meta-analysis achieved genome-wide significance. We then performed murine expression analyses to follow up this finding. Here, Isl1 expression was detected in the genital region within the critical time frame for human CBE development. Genital regions with *Isl1* expression included the peri-cloacal mesenchyme and the urorectal septum. The present study identified the first genome-wide significant locus for CBE at chromosomal region 5q11.1, and provides strong evidence for the hypothesis that *ISL1* is the responsible candidate gene in this region.

## Author Summary

The etiology of classic exstrophy of the bladder (CBE) remains unclear. The present genome-wide association study and meta-analysis identified an association between CBE and a region on chromosome 5q11.1. This region contains the gene encoding insulin gene enhancer protein, *ISL-1*. In this region, 138 single nucleotide polymorphisms (SNPs) reached genome-wide significance, with the SNP rs9291768 showing the lowest *P* value (p = 2.13 x 10^−12^). Our findings, as supported by expression analyses in murine models, suggest that *ISL1* is a susceptibility gene for CBE.

## Introduction

The bladder exstrophy-epispadias complex (BEEC; OMIM %600057) is the most severe of all human congenital anomalies of the kidney and urinary tract (CAKUT), and involves the abdominal wall, pelvis, all of the urinary tract, the genitalia, and occasionally the spine and anus. The severity-spectrum of the BEEC comprises the mildest form, epispadias (E); the intermediate form, classic bladder exstrophy (CBE); and the most severe form, exstrophy of the cloaca (CE) [1,2]. Despite advances in surgical techniques and improved understanding of the underlying anatomical defects, in later life many male and female patients experience chronic upper and lower urinary tract infections, sexual dysfunction, and urinary-, or in the case of cloacal exstrophies, urinary and fecal incontinence [3,4]. The estimated overall birth prevalence for the complete BEEC spectrum in children of European descent is 1 in 10 000 [5]. Birth prevalence, as assessed with the inclusion of terminated pregnancies, differs between subtypes. Estimated rates are: 1 in 117,000 in males and 1 in 484,000 in females for E [6]; 1 in 37,000 for CBE [6]; and 1 in 200,000 to 1 in 400,000 for CE [7]. According to the Birth Defects Monitoring Program of the Centers for Disease Control and Prevention, the birth prevalence of CBE among North American ethnic groups varies, with the highest birth prevalence being observed among Native Americans (8 in 100,000), and the lowest among Asians (1 in 100,000) [8]. Although BEEC can occur as part of a complex malformation syndrome, approximately 98.5% of cases are classified as isolated [9]. The reported recurrence risk for CBE among siblings in families with non-consanguineous and non-affected parents ranges between 0.3–2.3%, whereas the reported recurrence risk for the offspring of affected patients is 1.4% [10–12]. Hence, the recurrence risk for the offspring of CBE patients shows an approximate 400-fold increase compared to that observed in the general population [10]. Identification of genetic risk factors for the BEEC has been the subject of extensive recent research, and several lines of evidence support the hypothesis that genetic factors are implicated. These include reports of BEEC-associated chromosomal aberrations [13]; reports of at least 30 families with multiple affected members [13,14]; and observations of high concordance rates in monozygotic twins [5]. Array-based molecular karyotyping and regional association studies have implicated micro-duplications on chromosome 22q11.21 and polymorphisms in the *TP63* (*Tumor protein p63*) gene [15–19]. However, in the vast majority of cases, the genetic contribution to the BEEC remains elusive, and the molecular basis of the disruption of the respective developmental processes is poorly understood.

The aim of the present study was to identify susceptibility loci for CBE. Firstly, we conducted a genome-wide association study (GWAS) of 110 isolated CBE patients and 1,177 controls of European descent. Secondly, we performed a meta-analysis using the data from step 1 and data from our previous GWAS of 98 CBE patients and 526 controls [20]. Thirdly, we followed up our main finding by: (i) re-sequencing *ISL-1* (*ISL LIM homeobox 1*), the main candidate gene within the region of genome wide significance on chromosome 5q11.1, in all patients; and (ii) performing murine expression analyses.

## Results and Discussion

In the subsequent text, our previous GWAS [20] is termed GWAS1 and the present GWAS is termed GWAS2.

The post quality control data set of GWAS2 comprised 110 CBE patients and 1,177 controls. The GWAS2 analyses identified a region of approximately 220 kb on chromosome 5q11.1. This region harbors the gene *ISL1*. Multiple markers in this region showed evidence for association with CBE (S1 Table). The most significant marker, rs6874700, showed a *P* value of 6.27 x 10^−11^. The significance of this marker was supported by the presence of 172 surrounding markers with *P* values of < 10^−5^. A total of 84 markers at this locus, including rs6874700, reached genome-wide significance, i.e. P < 5 x 10^−8^. No other locus in the GWAS2 analyses achieved this level of significance.

**Fig 1:**
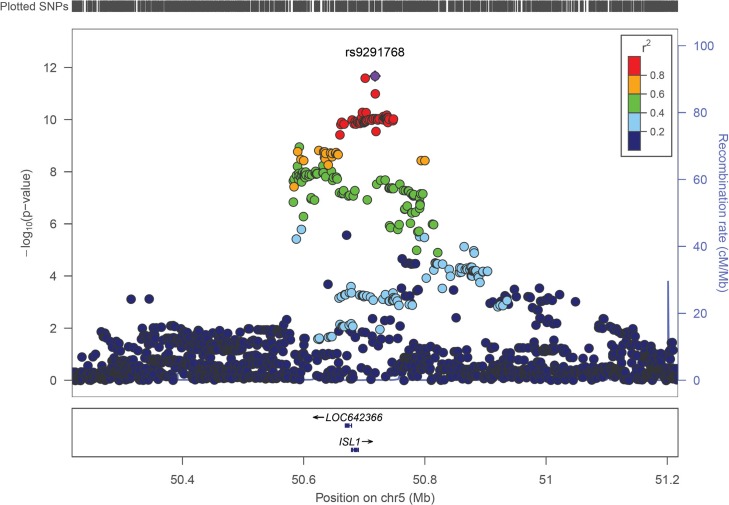
Regional association plot for *ISL1* across a 1.0 Mb window. Association with classic bladder exstrophy of individual SNPs in the meta-analysis GWAS is plotted as −log_10_(p) against chromosomal position. The y-axis on the right shows the recombination rate estimated from the 1000 Genomes (Mar 2012) EUR populations. All *P* values (y-axis on the left) are from the meta-analysis. The purple diamond indicates the most significant marker.

**Fig 2:**
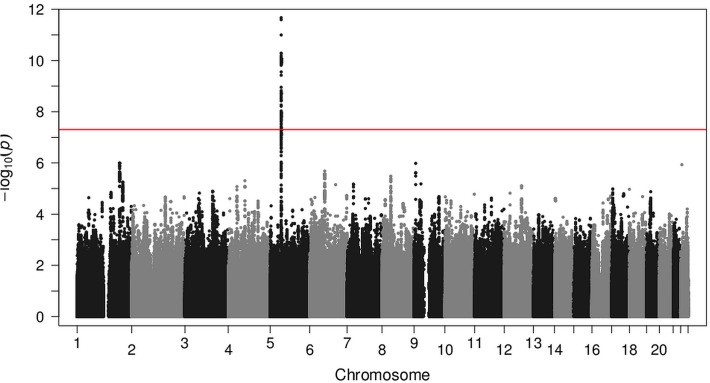
Genome-wide association scan in classic bladder exstrophy patients. Association of SNPs is plotted as −log_10_(p) against chromosomal position. The y-axis shows the negative log_10_ *P* values of the logistic regression for SNPs from the meta-analysis that passed quality control. Chromosomes are shown in alternating colors along the x-axis. The genome-wide significance level is indicated by a red line.

Next, we combined the effect estimates of GWAS1 and GWAS2 in a fixed effect meta-analysis. This meta-analysis also implicated the 220 kb region on chromosome 5q11.1. In the meta-analysis, multiple markers in this region showed evidence for association with CBE (Fig. 1). The most significant marker, rs9291768, had a *P* value of 2.13 x 10^−12^. The possible relevance of rs9291768 in CBE was supported by the presence of 137 surrounding markers with *P* values of < 5 x 10^−8^. No other locus in the meta-analysis achieved this level of significance (Fig. 2). All markers with *P* values of < 10^−5^ are listed in S2 Table.

The genotype-specific relative risks (RRs) for allele T of rs9291768 were: (i) RR_het = 2.00 for heterozygotes (95%-CI = 1.33–3.02); and (ii) RR_hom = 4.77 (95%-CI = 3.06–7.45) for homozygotes. This is compatible with neither a recessive (P = 3.9 x 10^−5^), nor a dominant mode of inheritance (*P* = 1.6 x 10^−4^). According to the genotype data from Ensembl release 74—December 2013©, the frequency of the CBE allele T at rs9291768 is highest in African populations (0.534), intermediate in European populations (0.425), and lowest in Asian populations (0.083).

Our previous study [20] failed to identify the possible relevance of both marker rs9291768 and the region comprising *ISL1*. In that report, rs9291768 obtained a *P* value of 1.1 x 10^−3^, which is not considered worthy of note in the context of a GWAS. In the present meta-analysis, the estimated relative risk for this SNP was 2.18 (see Table 1). The GWAS1 sample comprised 98 cases and 526 controls, and the power to achieve genome-wide significance (i.e. < 5 x 10^−8^) for a SNP with RR = 2.18 was only 31% under the assumption of a multiplicative model and a minor allele frequency of 0.377. For GWAS2, which comprised 110 cases and 1177 controls, the power was higher at 53%. However, the combination of GWAS1 and GWAS2 provides a power of 98%. This substantial increase in power is the central motivation for conducting meta-analyses. We therefore assume that the non-identification of rs9291768 in GWAS1 was attributable to the issues of power and random sample variation.

**Table 1:** Most strongly associated SNP in the bladder exstrophy susceptibility locus 5q11.1.

SNP	Chr	Position	Risk/other allel	RAF	RAF	RAF	RR [95% CI]	*P* value
				**cases**	**controls**	**combined**		
rs9291768	5	50717793	T/C	0.576	0.377	0.399	2.18 [1.75–2.71]	2.13 x 10^–12^

Relative risks (RRs) are given with the risk allele set as baseline. Chr, chromosome; RAF, risk allele frequency.

The marker rs9291768 is a non-coding variant, which is located 27.2 kb downstream of the *ISL1* gene. The associated 220 kb haplotype block contains the gene *ISL1*. The only other transcript encoded in the regions flanking rs9291768 (500 kb on either side) is LOC642366, which also resides within the associated haplotype block. LOC642366 encodes an uncharacterized non-coding RNA, which has no ortholog in mouse, zebrafish, drosophila, *C*. *elegans*, or *S*. *cerevisiae*. The second most proximal gene to rs9291768 is *Homo sapiens* poly (ADP-ribose) polymerase family, member 8 (*PARP8*), which is located 575 kb proximal to rs9291768. The third and fourth genes are *Homo sapiens* pelota homolog (Drosophila) (*PELO*), and the integrin, alpha 1 subunit of integrin receptors (*ITGA1*), which are both located ∼1.4 Mb distal to rs9291768. According to Mouse Genome Informatics (http://www.informatics.jax.org/), neither *PARP8* nor *ITGA1* is expressed in the genital tubercle or the cloacal membrane during the CBE critical time frame in mouse embryos. Furthermore, mice with complete invalidation of *PARP8* or *ITGA1* display neither CBE features nor CBE-related phenotypes. Studies of the Drosophila pelota gene have implicated *dPelo* in spermatogenesis, mitotic division, and patterning. Homozygous *Pelo*-null embryos fail to develop beyond embryonic day 7.5, and exhibit no early CBE-related features, such as diastases of the symphysis. Whether rs9291768 per se, or a variant in linkage disequilibrium with it, confers the functional effect underlying the association remains unclear. The rs9291768 marker shows no association with any predicted regulatory sequence (according to ENCODE, TFSEARCH, or FAS-ESS), or splicing motif. Of the other 136 markers at this chromosome 5 locus, only one (rs2303751) is located in a coding region, and none affect a splice site. Marker rs2303751 is in linkage disequilibrium (r^2^ = 0.932) with the most significant marker rs9291768, and represents a synonymous A/G substitution in exon four of *ISL1*. Furthermore, none of the public eQTL (expression quantitative trait loci) databases contains evidence to suggest that rs9291768, or a SNP in perfect linkage disequilibrium with it, would affect gene expression levels (RegulomeDB, http://regulome.stanford.edu; eQTL browser, eqtl.uchicago.edu).

The present meta-analysis generated no evidence in support of the (non-genome-wide significant) association between CBE and an intergenic region on chromosome 17q21.31-q21.32 identified in GWAS1 [20]. This region is located between the genes *WNT3* (wingless-type MMTV integration site family, member 3) and *WNT9b* (wingless-type MMTV integration site family, member 9b).

Since the CBE-associated region harbors the gene *ISL1*, we performed *ISL1* re-sequencing in 207 CBE patients included in the present meta-analysis. As well as allowing mutation detection, this approach should provide genotype data for polymorphisms in the exons and exon-flanking regions of *ISL1*. Using the results of Sanger sequencing, we compared genotype information from four SNPs with the imputed data. We calculated the allelic accuracy, i.e. the aggregate difference between the actual number of alleles observed and the number of imputed alleles [21]. This yielded accuracy values of 96.9% (rs150104955); 97.3% (rs2288468); 97.3% (rs2303751); and 99.5% (rs3917084). Two of these SNPs (rs2288468, rs2303751) achieved genome-wide significance in the meta-analysis

Although sequencing identified no nonsense or probably pathogenic *ISL1* variant, the following variants were all detected in a heterozygote state in single patients: intron 3, rs2303750; synonymous in exon 5, rs41268419 (p.Ser275 =); non-synonymous in exon 4, rs200209474 (p.Thr181Ser); unreported variants in intron 4, +21delG, -19delT, and -64A>G. Pathogenicity prediction using several publicly available algorithms (SNPs&Go, MutPred, SIFT) predicted that the p.Thr181Ser variant is neutral. Only PolyPhen-2 estimated it as possibly damaging. Furthermore, all of the observed intronic variants can be assumed to be benign. Hence, our patient sample size may have been too small to detect rare causal mutational events. We cannot exclude the possibility that some mutations were overlooked, i.e. mutations located in the promoter region, in as-yet-unknown regulatory sequences, or in non-coding regions that were not present within the covered sequence.

*ISL1* encodes the insulin gene enhancer protein ISL1, a LIM zinc-binding/homeobox-domain transcription factor which was initially identified as a regulator of insulin expression [22]. Research in rodents suggests that Isl1 plays a fundamental role in the embryogenesis of multiple tissue types: Isl1 affects cell differentiation and survival, cell fate determination, the generation of cell diversity, and segmental patterning during mouse development [23]. Isl1 binds and regulates the promoters of the glucagon and somatostatin genes, and activates insulin gene transcription in pancreatic beta cells in synergy with *NEUROD1* (neuronal differentiation 1) [24]. A previous study found an association between a heterozygous *ISL1* premature termination mutation (p.Gln310*) and diabetes type II in a large Japanese kindred [25]. Furthermore, in a classic linkage analysis of 186 Swedish multiplex families with diabetes type I, linkage was observed with chromosomal region 5q11-q13, which harbors *ISL1* [26]. This finding supports the hypothesis that ISL1 is implicated in pancreatic function and development, as reported in *Isl1* knockout mice [27]. In the mouse, research at E (embryonic day) 8.5 to E9.5 has shown that ISL1 acts upstream of the sonic hedgehog (Shh) signaling pathway [28], which may be involved in other processes besides the coordination of heart and lung co-development [29]. Interestingly, a recent report by Matsumaru et al. [30] showed that SHH is also important for ventral body wall formation, and that ectopic SHH signaling induces omphalocele, a feature which is associated with CE, the severest form of the BEEC.

A previous study in mice also showed that a homozygous *Isl1* null mutation (*Isl1* ^-/-^) induced growth retardation at E9.5 and severe cardiac malformations at E10.5 [31]. Embryos exhibiting these severe cardiac malformations at E10.5 died at E11.5 due to the developmental arrest of spinal motor neurons [32]. Research has demonstrated a further role for Isl1 in mice at E11.5, i.e. in hindlimb-specific patterning and growth in combination with both SHH and the helix-loop-helix transcription factor HAND2 (heart and neural crest derivatives expressed 2) [33]. This interplay is also necessary for normal cardiac development in mice [23]. Recently, Jurberg et al. reported that specific activity of mouse *Isl1* in the progenitors of the ventral lateral mesoderm promotes formation of the cloaca-associated mesoderm as the most posterior derivatives of lateral mesoderm progenitors [34]. This observation provides independent evidence that *ISL1* is a promising candidate gene for human CBE.

In a recent mouse study, Kaku et al. induced conditional *Isl1* deletion in the lateral mesoderm using a *Hoxb6-Cre* driver, and demonstrated that this caused kidney agenesis or hydroureter [35]. The authors observed transient *Isl1* expression between E10.5—E14.5. At early stages, this was observed in the mesenchyme surrounding the ureteric stalk and cloaca. At later stages, expression occurred along the nephric duct, at the base of the ureteric stalk, and in the genital tubercle. This suggests that *Isl1* may be implicated in kidney, ureter, and bladder development. These mice show a variable phenotype, which can include agenesis of the genital tubercle (R. Nishinakamura, personal communication). The variability of this defect is probably due to mosaicism, which arises as a result of the *Hoxb6-Cre* driver [33]. Kaku et al. also reported that conditional loss of *Isl1* resulted in a concomitant reduction in the expression of bone morphogenetic protein 4 (Bmp4) [35]. Using mouse *Isl1Cre;Bmp4* ^*flox/flox*^ mutants, Suzuki et al. showed that BMP4 signaling in the caudal *Isl1* expression domain was required for formation of the anterior peri-cloacal mesenchyme (aPCM) at E10.5 [36]. Rather than decreasing Isl1 function, loss of this signal caused defective pelvic and urogenital organ formation, including kidney and bladder agenesis, with abnormal development of the lower limbs and pelvis. Moreover, tissue lineage analyses suggested that *Isl1*-expressing cells are an essential cell population in terms of caudal body formation, including the pelvic/urogenital organs and hindlimb [36].

**Fig 3:**
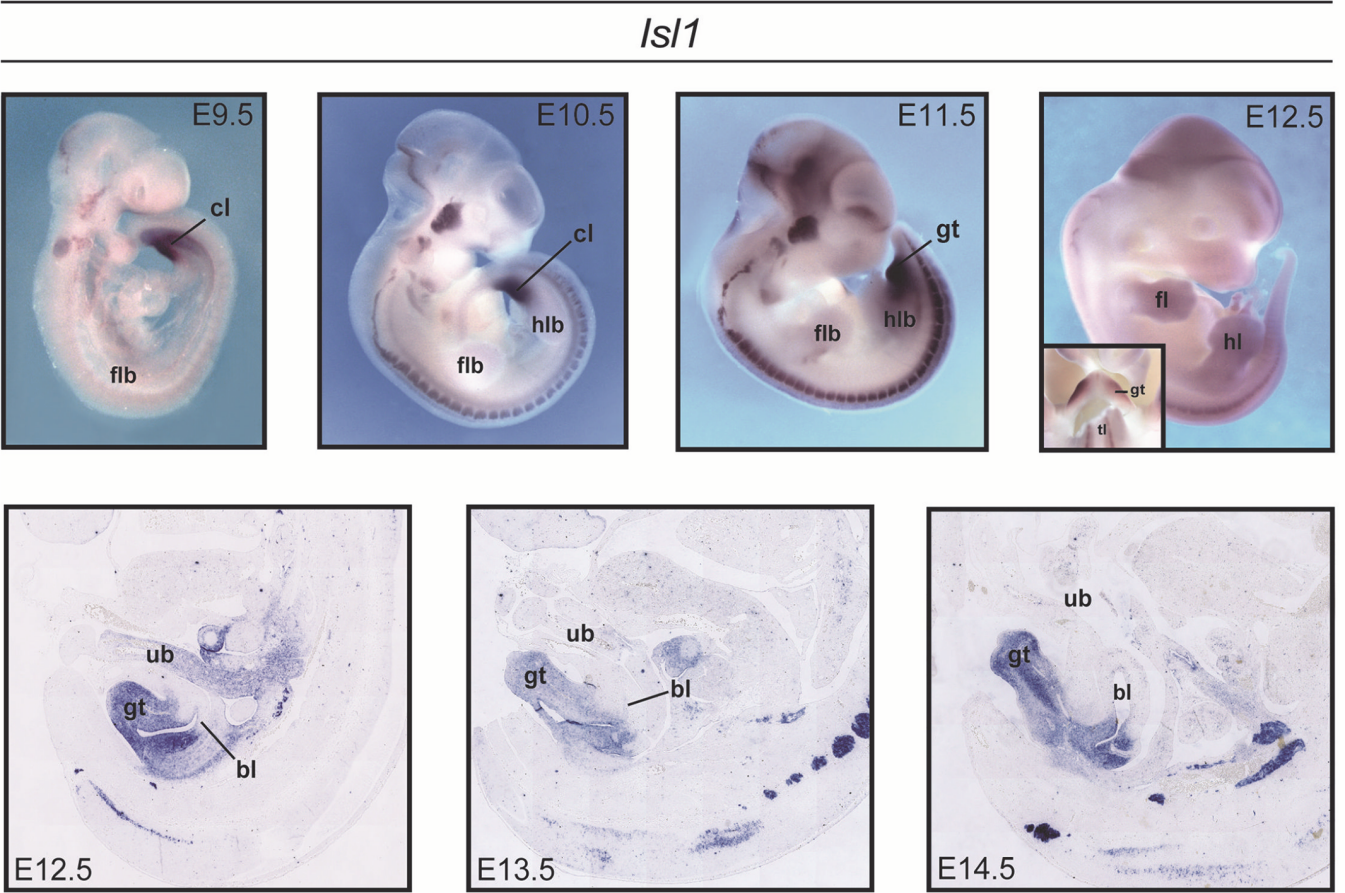
Expression of *Isl1* during mouse development. Whole-mount *in situ* hybridization (ISH) for *Isl1* in wildtype mouse embryos between E9.5-E12.5 revealed strong expression in the developing genital region, including the cloaca, cloacal membrane, and emerging genital tubercle. ISH on mid-sagittal paraffin sections at later embryonic stages (E12.5-E14.5) revealed expression throughout the genital tubercle, within the peri-cloacal mesenchyme and urorectal septum. *Isl1* was also detected in the craniofacial- and spinal ganglia.

In the present mouse analyses, *Isl1* was expressed during the critical timeframe for development of tissues involved in CBE, and strong *Isl1* expression was detected in the developing genital region (Fig. 3). From E9.5, a broad *Isl1* domain was detected in the cloacal region. This was maintained in the outgrowing genital tubercle (including the urorectal septum) until at least E14.5.

Three groups have recently elucidated the molecular basis of the Danforth’s short tail (*Sd*) mouse. They reported the insertion of a retrotransposon in the 5’ regulatory domain of the murine *Ptf1a* gene, which encodes pancreas specific transcription factor 1A [37–39]. As a consequence, and in contrast to their wildtype littermates, *Sd* mice showed ectopic *Ptf1a* expression in the notochord and hindgut at E8.5 to E9.5, which extended to the cloaca and mesonephros at E10.5 and to the pancreatic bud at E10.5 and E11.5 [38]. The resultant phenotype of this *Sd* mutation mirrors the phenotype observed in human caudal malformation syndromes, a phenotype that is also observed in *Isl1* transgenic mice [40]. Moreover, the BEEC related human Currarino syndrome (MIM: #176450), which comprises hemisacrum, anorectal malformations, and a presacral mass, is caused by mutations of the transcription factor *MNX1*/*HLXB9* (Motor neuron and pancreas homeobox protein 1). The genes *Ptf1a*, *Isl1*, and *Mnx1* have been implicated in pancreas development, and MNX1 has been identified as a direct target of PTF1a [41].

Coordinated development of caudal body structures is necessary for the formation of the bladder, rectum, and the external genitalia [42,43]. These organs are derived from the transient embryonic cloaca and the PCM, an infra-umbilical mesenchyme [44], as well as the anterior PCM [36,42]. SHH-, ISL1-, and BMP4-expressing cells contribute to both the PCM and the anterior PCM [36,42,45]. Perturbation of this morphoregulatory network may thus lead to malformation of caudal structures, including the bladder, rectum, and external genitalia.

In summary, the present report describes a novel association between *ISL1* and human CBE. While previous conventional linkage- and candidate gene studies in humans have suggested the involvement of *ISL1* in diabetes type I and II and in congenital heart defects, to our knowledge, the present study is the first to implicate *ISL1* in the formation of human urogenital malformations. The observed variation in CBE birth prevalence across populations is consistent with the cross-population frequencies of the rs9291768 T-allele, thus supporting our finding.

The importance of *Pft1a* and *Isl1* in the formation of murine genital development and caudal regression phenotypes, the involvement of *MNX1* in the BEEC related human Currarino syndrome, and the role of all three genes in pancreatic development suggest that these genes are involved in a common pathway. However, the present data do not exclude the possibility that the association between CBE and the region surrounding *ISL1* is attributable to long-range functional interactions with other regions in the human genome. Future studies are warranted to identify the mechanisms through which genetic variation at *ISL1* contributes to CBE development.

## Methods

### Subjects GWAS2

The initial GWAS2 sample comprised 123 isolated CBE patients and 1,320 controls of European descent. Prior to inclusion, written informed consent was obtained from all subjects, or from their proxies in the case of legal minors. For patients and controls, demographic information was collected using a structured questionnaire. This study was approved by the institutional ethics committee of each participating center, and was conducted in accordance with the principles of the Declaration of Helsinki. All CBE patients were recruited in person by experienced physicians trained in the diagnosis of the BEEC. Details of the recruitment process for patients and controls are provided in Reutter et al. [20].

### Genotyping

For the 123 isolated CBE patients in GWAS2, genotyping was performed using the Illumina BeadChip HumanOmniExpress (San Diego, California, USA), and DNA was extracted from blood or saliva using standard procedures. Case-control comparisons were made using the genotypes of 1,320 population-based controls, which had been processed using the same array [46]. Genome-wide genotyping of 730,525 markers was conducted using the Infinium HD Ultra Assay from Illumina (Illumina, San Diego, California, USA).

### Pre-imputation quality control of GWAS2

Markers were excluded from the analysis if: (i) the minor allele frequency was <1% or the call rate was <95% in either cases or controls; or (ii) the test for Hardy-Weinberg equilibrium resulted in *P*<10^−4^ in the control sample or *P*<10^−6^ in the case sample. A total of 616,799 autosomal markers fulfilled these quality criteria. Individuals were excluded if their call rate was <99%, or if they were outliers in a multidimensional scaling (MDS) analysis. Relatedness of individuals within GWAS2, and between GWAS1 and GWAS2, was evaluated using both the KING program [47], and an identity-by-state-based in-house program. The post quality control data set of GWAS2 comprised 110 CBE patients and 1,177 controls.

### Imputation

GWAS1 and GWAS2 were imputed separately to the 1000 Genomes Project and HapMap 3 reference panels using IMPUTE2 [48].

### Post-imputation quality control

For each of the three data sets, variants were excluded if: (i) the imputation info score was <0.4; (ii) the dosage of the minor allele was <1% in either cases or controls; (iii) the test for Hardy-Weinberg equilibrium (calculated on the basis of the 80% best-guess genotypes) resulted in *P*<10^−4^ in the control sample; or (iv) the 80% best-guess genotypes were only available for <80% of cases or controls. In total, 7,261,187 SNPs were analyzed in at least one data set.

### Statistical analysis

Single-marker analysis was performed using logistic regression. The allele dosage and the first five components obtained from MDS were used as independent variables for the variants in the three data sets. The effect estimates for the data sets were then combined in an inverse variance-weighted fixed-effects meta-analysis. The genomic inflation factor in this meta-analysis was 1.0196.

### Power

Power was calculated to enable detection of genome-wide significance (*P* <5 x 10^−8^) in the combined analysis of the GWAS1 and GWAS2 samples. Under the assumption of a multiplicative model, this was 80% for an allele frequency of 0.35 (0.20) and a RR of 1.94 (2.05). This is within the range of RRs observed for other multifactorial, nonsyndromic human malformations. For example, the power of the present study to detect a locus with an effect-strength similar to that of the most strongly associated locus in nonsyndromic cleft lip with or without cleft palate was 99.2% [49].

### *ISL-1* resequencing

Sequence analysis of the complete *ISL-1* coding regions and their splice consensus motifs was performed in 207 of our 208 CBE patients using standard techniques. Primers are listed in S3 Table. For the remaining patient, no additional DNA sample was available. During this analysis, we also obtained information for several SNPs deposited in dbSNP Build142 (rs3917084, rs150104955, rs2288468, rs2303750, rs2303751, rs200209474, and rs41268419).

### In situ hybridization of mouse embryo sections

The expression of Isl1 was analyzed using in situ hybridization, standard procedures, and a ∼450bp antisense probe spanning exons 2 and 3 from XM_006517533.1. Details of the in situ hybridization methods are provided elsewhere [50].

## Supporting Information

S1 Table All markers identified in GWAS 2 within chromosomal region 5q11.1 with *P* values < 10^−5^. The most significant marker, rs6874700, showed a *P* value of 6.27 x 10^−11^. Relative risks (RRs) are given with the risk allele set as baseline. Chr, chromosome; RAF, risk allele frequency.
(PDF)

S2 Table All markers identified in the meta-analysis of GWAS 1 and GWAS 2 over the entire genome with *P* values of < 10^−5^. Relative risks (RRs) are given with the risk allele set as baseline. Chr, chromosome; RAF, risk allele frequency.
(PDF)

S3 Table Primers (5’->3’ direction) used for *ISL1* sequence analysis. F, forward; R, reverse.
(PDF)
