# Supplementary material for: Genome-wide Association Study and Meta-Analysis Identify ISL1 as Genome-wide Significant Susceptibility Gene for Bladder Exstrophy
Source: PLoS Genet. 2015 Mar 12;11(3):e1005024. doi: 10.1371/journal.pgen.1005024 (PMC4357422; doi:10.1371/journal.pgen.1005024)
Supplement: S1 Table — The most significant marker, rs6874700, showed a P value of 6.27 x 10^−11^. Relative risks (RRs) are given with the risk allele set as baseline. Chr, chromosome; RAF, risk allele frequency. (PDF) [file pgen.1005024.s001.pdf]

Supplementary Table 1. GWAS2 results in classic bladder exstrophy

SNP	Chr	Position	Risk/ other allel	RAF cases	RAF controls	RAF combined	RR [95% CI]	*P* value	Imputed
rs6874700	5	50701750	A/T	0.605	0.362	0.383	2.62 [1.96–3.49]	6.27E-11	I
rs9291768	5	50717793	T/C	0.605	0.364	0.384	2.60 [1.95–3.47]	8.89E-11	I
rs4865663	5	50702964	T/C	0.600	0.369	0.389	2.52 [1.89–3.37]	3.30E-10	I
rs4865658	5	50680233	A/G	0.604	0.376	0.395	2.52 [1.89–3.37]	4.20E-10	I
rs991216	5	50688533	G/C	0.604	0.374	0.394	2.51 [1.88–3.36]	4.27E-10	I
rs6861333	5	50703933	G/C	0.607	0.377	0.397	2.52 [1.88–3.36]	4.39E-10	I
rs28664909	5	50706499	A/G	0.605	0.376	0.396	2.51 [1.88–3.35]	4.40E-10	I
rs6449612	5	50701102	T/C	0.605	0.376	0.395	2.51 [1.88–3.35]	4.40E-10	I
rs973860	5	50701139	T/C	0.605	0.376	0.395	2.51 [1.88–3.35]	4.40E-10	I
rs1158641	5	50703285	A/C	0.607	0.377	0.397	2.51 [1.88–3.36]	4.50E-10	I
rs6449610	5	50698339	C/T	0.605	0.375	0.395	2.51 [1.88–3.35]	4.59E-10	I
rs991217	5	50688801	A/G	0.605	0.375	0.395	2.50 [1.88–3.34]	4.65E-10	I
rs2303751	5	50685505	G/A	0.605	0.375	0.395	2.50 [1.88–3.34]	4.67E-10	I
rs10076515	5	50702024	A/G	0.606	0.377	0.397	2.51 [1.88–3.35]	4.70E-10	I
rs10040820	5	50694579	G/A	0.605	0.375	0.395	2.50 [1.88–3.34]	4.71E-10	I
rs10041392	5	50700597	G/A	0.606	0.377	0.396	2.51 [1.88–3.35]	4.79E-10	I
rs6449622	5	50709369	G/C	0.606	0.377	0.397	2.51 [1.88–3.36]	4.83E-10	I
rs6895297	5	50695877	A/G	0.605	0.375	0.395	2.50 [1.87–3.34]	4.91E-10	-
rs6449609	5	50696967	A/T	0.607	0.380	0.399	2.51 [1.88–3.36]	5.02E-10	I
rs1017	5	50690095	T/A	0.606	0.376	0.396	2.50 [1.87–3.34]	5.15E-10	I
rs4865662	5	50699552	G/A	0.607	0.378	0.397	2.50 [1.87–3.35]	5.17E-10	I
rs6861877	5	50691723	C/G	0.606	0.376	0.396	2.50 [1.87–3.34]	5.18E-10	I
rs7713979	5	50714556	G/A	0.605	0.377	0.397	2.50 [1.87–3.35]	5.41E-10	I
rs7709134	5	50693176	A/G	0.606	0.377	0.397	2.50 [1.87–3.34]	5.49E-10	I
rs9291766	5	50715254	A/G	0.605	0.377	0.397	2.50 [1.87–3.34]	5.64E-10	I
rs6449623	5	50712332	G/C	0.606	0.378	0.398	2.50 [1.87–3.34]	5.94E-10	I
rs6859394	5	50686582	C/T	0.606	0.378	0.398	2.50 [1.87–3.33]	6.26E-10	I
rs2288468	5	50683655	C/T	0.605	0.377	0.396	2.48 [1.86–3.31]	6.35E-10	-
rs2115322	5	50666618	G/A	0.602	0.376	0.396	2.49 [1.86–3.33]	6.89E-10	I
rs10454819	5	50718216	A/G	0.585	0.360	0.379	2.49 [1.86–3.32]	6.95E-10	I
rs4481304	5	50759415	A/G	0.648	0.420	0.440	2.54 [1.89–3.42]	7.02E-10	I
rs4865512	5	50661601	G/A	0.601	0.376	0.395	2.49 [1.86–3.32]	7.22E-10	I
rs10454820	5	50718769	A/G	0.606	0.379	0.398	2.49 [1.86–3.33]	7.28E-10	I
rs10039350	5	50737297	C/T	0.604	0.377	0.396	2.48 [1.86–3.30]	7.45E-10	I
rs6869470	5	50663015	T/C	0.597	0.372	0.391	2.47 [1.85–3.30]	7.49E-10	I
rs10939960	5	50738447	T/C	0.605	0.379	0.398	2.47 [1.85–3.30]	8.15E-10	I
rs4865666	5	50737629	T/A	0.605	0.379	0.398	2.47 [1.85–3.30]	8.19E-10	I
rs10066455	5	50721742	A/G	0.605	0.380	0.399	2.48 [1.86–3.31]	8.35E-10	I
rs7717071	5	50739735	T/C	0.604	0.378	0.398	2.47 [1.85–3.30]	8.38E-10	I
rs10939961	5	50738478	A/C	0.604	0.378	0.397	2.47 [1.85–3.30]	8.40E-10	I
rs13356912	5	50738088	G/A	0.604	0.378	0.397	2.47 [1.85–3.30]	8.42E-10	I
rs11951998	5	50736314	C/T	0.605	0.379	0.398	2.47 [1.85–3.30]	8.43E-10	-
rs7714459	5	50731536	C/T	0.603	0.377	0.396	2.47 [1.85–3.30]	8.46E-10	I
rs10041557	5	50734335	G/C	0.604	0.378	0.397	2.47 [1.85–3.30]	8.56E-10	I
rs10040678	5	50737159	A/G	0.604	0.378	0.397	2.47 [1.85–3.29]	8.69E-10	I
rs4865667	5	50748173	T/C	0.605	0.379	0.399	2.48 [1.86–3.32]	8.69E-10	-
rs10939954	5	50724774	G/A	0.604	0.378	0.397	2.47 [1.85–3.30]	8.72E-10	I
rs7733960	5	50735905	T/C	0.604	0.378	0.397	2.47 [1.85–3.29]	8.73E-10	I
rs10066521	5	50721825	T/G	0.604	0.378	0.398	2.47 [1.85–3.30]	8.81E-10	I
rs10043658	5	50733422	G/A	0.604	0.378	0.398	2.47 [1.85–3.29]	8.85E-10	I
rs6891088	5	50734414	C/T	0.604	0.378	0.398	2.47 [1.85–3.29]	8.89E-10	I

SNP	Chr	Position	Risk/ other allel	RAF cases	RAF controls	RAF combined	RR [95% CI]	*P* value	Imputed
rs6865525	5	50734787	A/G	0.604	0.378	0.398	2.47 [1.85–3.29]	8.90E-10	I
rs62368261	5	50722381	A/G	0.604	0.379	0.398	2.47 [1.85–3.30]	9.07E-10	I
rs4865664	5	50722238	C/A	0.605	0.379	0.399	2.47 [1.85–3.30]	9.28E-10	-
rs7721741	5	50741017	C/G	0.605	0.380	0.399	2.48 [1.85–3.31]	9.31E-10	I
rs7714281	5	50731380	A/T	0.605	0.380	0.399	2.46 [1.85–3.29]	1.01E-09	I
rs10039723	5	50739152	C/A	0.605	0.381	0.400	2.46 [1.84–3.29]	1.15E-09	-
rs7705789	5	50737991	A/G	0.597	0.375	0.394	2.46 [1.84–3.28]	1.22E-09	I
rs10939963	5	50747502	C/A	0.609	0.384	0.404	2.44 [1.83–3.27]	1.56E-09	I
rs9291770	5	50719408	G/T	0.592	0.375	0.393	2.42 [1.81–3.24]	2.35E-09	I
rs6449608	5	50696922	A/T	0.571	0.359	0.378	2.39 [1.80–3.19]	2.78E-09	I
rs4865656	5	50659788	A/G	0.586	0.371	0.389	2.38 [1.78–3.16]	3.17E-09	-
rs1423611	5	50771114	T/G	0.759	0.541	0.560	2.67 [1.93–3.71]	3.64E-09	-
rs1364000	5	50767349	A/G	0.760	0.544	0.562	2.65 [1.91–3.68]	5.16E-09	I
rs2161593	5	50766915	A/T	0.760	0.544	0.563	2.65 [1.91–3.68]	5.24E-09	I
rs2059223	5	50778482	A/T	0.757	0.546	0.564	2.59 [1.87–3.59]	9.89E-09	I
rs17824958	5	50779044	A/G	0.757	0.546	0.564	2.59 [1.87–3.58]	1.04E-08	I
rs6891554	5	50784546	A/C	0.755	0.545	0.563	2.58 [1.86–3.57]	1.06E-08	-
rs12332270	5	50776171	T/C	0.746	0.540	0.557	2.52 [1.83–3.48]	1.80E-08	I
rs4343805	5	50648159	T/C	0.581	0.386	0.403	2.37 [1.76–3.21]	2.05E-08	I
rs6872771	5	50653635	C/T	0.623	0.421	0.438	2.28 [1.70–3.05]	3.36E-08	I
rs2405154	5	50625038	G/A	0.623	0.419	0.437	2.26 [1.69–3.02]	3.62E-08	I
rs7349819	5	50590195	T/G	0.627	0.423	0.440	2.27 [1.69–3.04]	3.65E-08	I
rs6449585	5	50632997	G/A	0.623	0.419	0.437	2.25 [1.69–3.01]	3.78E-08	I
rs6414789	5	50634836	A/T	0.623	0.420	0.437	2.25 [1.69–3.01]	3.78E-08	I
rs6414788	5	50634777	C/T	0.621	0.418	0.436	2.26 [1.69–3.02]	3.80E-08	I
rs7709389	5	50656032	C/G	0.618	0.418	0.435	2.26 [1.69–3.03]	4.23E-08	I
rs2217316	5	50656853	A/G	0.618	0.418	0.435	2.26 [1.69–3.03]	4.24E-08	I
rs7702670	5	50636650	T/C	0.623	0.421	0.438	2.25 [1.68–3.01]	4.28E-08	-
rs6881501	5	50652823	A/G	0.619	0.419	0.436	2.26 [1.69–3.03]	4.28E-08	I
rs6867206	5	50652694	C/T	0.618	0.418	0.435	2.26 [1.69–3.02]	4.30E-08	I
rs10939913	5	50592825	C/G	0.427	0.249	0.265	2.26 [1.69–3.02]	4.33E-08	I
rs2195406	5	50644455	G/C	0.618	0.418	0.435	2.26 [1.69–3.02]	4.39E-08	I
rs10037522	5	50635643	C/A	0.623	0.421	0.438	2.24 [1.68–3.00]	4.64E-08	-
rs9687742	5	50647350	C/T	0.615	0.418	0.435	2.25 [1.68–3.01]	5.57E-08	I
rs10050915	5	50791335	T/C	0.769	0.573	0.590	2.48 [1.78–3.45]	7.11E-08	I
rs10755219	5	50601746	C/T	0.429	0.254	0.269	2.25 [1.68–3.02]	7.34E-08	I
rs9687615	5	50647257	C/A	0.613	0.417	0.434	2.23 [1.66–2.99]	7.43E-08	I
rs1501985	5	50595433	C/T	0.616	0.419	0.435	2.20 [1.65–2.94]	9.08E-08	I
rs6449543	5	50590024	C/T	0.439	0.264	0.279	2.20 [1.65–2.94]	9.25E-08	I
rs10066412	5	50600177	T/A	0.614	0.418	0.435	2.19 [1.64–2.93]	1.05E-07	I
rs9291769	5	50717827	G/A	0.560	0.383	0.398	2.25 [1.66–3.04]	1.58E-07	I
rs6449586	5	50634878	T/C	0.745	0.559	0.575	2.37 [1.72–3.28]	1.58E-07	-
rs28454398	5	50793259	G/T	0.652	0.458	0.474	2.19 [1.63–2.95]	1.74E-07	I
rs7737645	5	50796827	C/G	0.652	0.458	0.475	2.19 [1.63–2.94]	1.76E-07	I
rs7732492	5	50786412	G/A	0.750	0.564	0.580	2.36 [1.71–3.26]	1.91E-07	I
rs4141541	5	50584237	C/T	0.623	0.434	0.450	2.18 [1.63–2.93]	1.93E-07	I
rs1501987	5	50591998	T/C	0.427	0.259	0.273	2.17 [1.62–2.90]	1.96E-07	I
rs7700319	5	50598539	C/T	0.427	0.259	0.274	2.16 [1.62–2.89]	2.12E-07	I
rs10036382	5	50612558	C/T	0.432	0.264	0.279	2.14 [1.60–2.87]	2.93E-07	-
rs10067551	5	50640597	C/A	0.623	0.438	0.454	2.14 [1.60–2.86]	3.36E-07	I
rs2113077	5	50799442	G/A	0.751	0.567	0.583	2.33 [1.68–3.22]	3.60E-07	I
rs148490291	5	50604419	T/C	0.435	0.270	0.284	2.15 [1.60–2.88]	3.71E-07	I

SNP	Chr	Position	Risk/ other allel	RAF cases	RAF controls	RAF combined	RR [95% CI]	*P* value	Imputed
rs1423618	5	50800179	C/T	0.541	0.363	0.378	2.08 [1.57–2.77]	4.02E-07	I
rs1559278	5	50794221	C/T	0.541	0.364	0.379	2.08 [1.57–2.76]	4.09E-07	-
rs2033237	5	50596784	C/T	0.427	0.264	0.278	2.11 [1.58–2.82]	5.18E-07	-
rs963194	5	50618895	G/C	0.343	0.200	0.212	2.18 [1.60–2.97]	8.05E-07	I
rs6449584	5	50632719	C/T	0.741	0.573	0.588	2.21 [1.61–3.05]	1.28E-06	I
rs10077073	5	50631992	A/G	0.357	0.214	0.226	2.08 [1.54–2.81]	1.74E-06	I
rs16875094	5	50734670	G/A	0.405	0.251	0.264	2.01 [1.51–2.68]	2.09E-06	-
rs10045426	5	50619895	A/C	0.355	0.213	0.225	2.07 [1.53–2.80]	2.11E-06	I
rs16878735	5	50598614	T/A	0.348	0.208	0.220	2.08 [1.54–2.82]	2.32E-06	I
rs72499502	5	50603530	A/G	0.349	0.207	0.219	2.06 [1.53–2.78]	2.34E-06	I
rs142461099	5	50602739	T/G	0.347	0.206	0.218	2.07 [1.53–2.79]	2.38E-06	I
rs142059785	5	50602256	G/C	0.349	0.207	0.219	2.06 [1.52–2.78]	2.40E-06	I
rs11745678	5	50609478	G/C	0.353	0.211	0.223	2.06 [1.53–2.78]	2.41E-06	I
rs963195	5	50618929	G/C	0.354	0.212	0.224	2.06 [1.53–2.78]	2.43E-06	I
rs10038485	5	50596639	T/A	0.354	0.211	0.224	2.06 [1.52–2.78]	2.45E-06	I
rs10059280	5	50612871	A/G	0.353	0.211	0.223	2.06 [1.52–2.78]	2.49E-06	I
rs143466268	5	50604309	T/C	0.354	0.211	0.224	2.06 [1.52–2.78]	2.49E-06	I
rs9291739	5	50608685	A/G	0.354	0.212	0.224	2.06 [1.52–2.78]	2.50E-06	I
rs72761990	5	50599662	A/G	0.354	0.212	0.224	2.06 [1.52–2.78]	2.51E-06	I
rs10071749	5	50601535	C/T	0.354	0.212	0.224	2.06 [1.52–2.78]	2.54E-06	I
rs7711781	5	50593076	T/C	0.354	0.212	0.224	2.06 [1.52–2.77]	2.55E-06	I
rs10067157	5	50645956	G/A	0.350	0.209	0.221	2.06 [1.52–2.78]	2.56E-06	I
rs6449541	5	50588359	T/C	0.354	0.212	0.224	2.05 [1.52–2.77]	2.60E-06	I
rs10040324	5	50632741	A/C	0.354	0.213	0.225	2.06 [1.52–2.78]	2.62E-06	I
rs10039627	5	50632050	T/C	0.353	0.213	0.225	2.06 [1.52–2.78]	2.69E-06	I
rs9291746	5	50622094	T/C	0.353	0.212	0.224	2.05 [1.52–2.78]	2.70E-06	I
rs10471584	5	50759020	A/G	0.401	0.251	0.264	2.00 [1.50–2.68]	2.74E-06	I
rs2217314	5	50622582	C/T	0.353	0.213	0.225	2.05 [1.52–2.77]	2.76E-06	I
rs10939957	5	50727004	A/G	0.394	0.246	0.259	2.01 [1.50–2.69]	2.92E-06	I
rs28557169	5	50720443	A/T	0.400	0.250	0.263	1.99 [1.49–2.66]	2.95E-06	I
rs55867625	5	50599470	G/A	0.338	0.202	0.214	2.10 [1.54–2.87]	2.95E-06	I
rs10038557	5	50620779	C/T	0.354	0.213	0.225	2.05 [1.52–2.76]	3.00E-06	I
rs10052407	5	50591476	T/C	0.355	0.213	0.225	2.04 [1.51–2.75]	3.22E-06	-
rs10055766	5	50654382	G/A	0.353	0.213	0.225	2.04 [1.51–2.76]	3.35E-06	I
rs2897069	5	50654438	G/A	0.353	0.213	0.225	2.04 [1.51–2.76]	3.35E-06	I
rs9291757	5	50653781	A/G	0.353	0.214	0.225	2.04 [1.51–2.76]	3.36E-06	I
rs9291732	5	50584783	G/C	0.351	0.212	0.224	2.04 [1.51–2.76]	3.52E-06	I
rs17398816	5	50595723	T/C	0.350	0.210	0.222	2.04 [1.51–2.75]	3.54E-06	I
rs144781991	5	50649357	T/G	0.352	0.213	0.225	2.04 [1.51–2.76]	3.57E-06	I
rs1423617	5	50801564	A/G	0.385	0.241	0.253	2.01 [1.50–2.71]	3.59E-06	I
rs9291760	5	50655574	T/C	0.352	0.213	0.225	2.04 [1.51–2.75]	3.71E-06	I
rs9291750	5	50636521	G/T	0.355	0.215	0.227	2.03 [1.50–2.74]	3.81E-06	-
rs10075310	5	50649562	G/A	0.341	0.206	0.218	2.07 [1.52–2.81]	4.10E-06	I
rs12521818	5	50583089	T/C	0.352	0.214	0.226	2.03 [1.50–2.75]	4.11E-06	I
rs7720488	5	50746191	A/G	0.399	0.251	0.264	1.97 [1.48–2.64]	4.24E-06	I
rs4865668	5	50748359	G/A	0.399	0.251	0.264	1.97 [1.48–2.64]	4.25E-06	I
rs7722654	5	50740885	T/C	0.395	0.248	0.261	1.98 [1.48–2.64]	4.27E-06	I
rs9291777	5	50742575	T/C	0.399	0.251	0.264	1.97 [1.48–2.63]	4.30E-06	I
rs60195170	5	50745323	T/C	0.395	0.248	0.260	1.98 [1.48–2.64]	4.33E-06	I
rs10471549	5	50665962	G/A	0.393	0.247	0.259	1.96 [1.47–2.61]	4.67E-06	I
rs10063466	5	50647670	T/C	0.350	0.213	0.225	2.02 [1.50–2.73]	4.76E-06	I
rs9885436	5	50764649	A/G	0.399	0.252	0.264	1.96 [1.47–2.62]	4.76E-06	I

SNP	Chr	Position	Risk/ other allel	RAF cases	RAF controls	RAF combined	RR [95% CI]	*P* value	Imputed
rs7448043	5	50768011	C/T	0.398	0.251	0.264	1.96 [1.47–2.62]	4.80E-06	I
rs11954894	5	50674131	T/C	0.395	0.248	0.261	1.95 [1.46–2.60]	5.02E-06	I
rs3811911	5	50684451	T/C	0.395	0.248	0.261	1.95 [1.46–2.60]	5.05E-06	-
rs2161592	5	50772554	T/C	0.398	0.251	0.264	1.96 [1.47–2.61]	5.17E-06	I
rs10056724	5	50665516	G/A	0.395	0.249	0.261	1.95 [1.46–2.60]	5.27E-06	I
rs10075800	5	50659190	A/C	0.395	0.249	0.261	1.95 [1.46–2.60]	5.33E-06	I
rs2161591	5	50772920	A/G	0.398	0.251	0.264	1.95 [1.46–2.61]	5.42E-06	I
rs10055984	5	50663550	T/C	0.395	0.249	0.261	1.95 [1.46–2.60]	5.44E-06	I
rs11747876	5	50773488	T/A	0.398	0.252	0.264	1.95 [1.46–2.61]	5.45E-06	I
rs3792733	5	50682065	A/G	0.396	0.250	0.262	1.95 [1.46–2.59]	5.50E-06	I
rs6867226	5	50705034	T/C	0.397	0.252	0.264	1.95 [1.46–2.60]	5.79E-06	I
rs7708866	5	50676126	C/T	0.395	0.249	0.262	1.94 [1.46–2.59]	5.90E-06	I
rs2059224	5	50777596	A/G	0.398	0.254	0.267	1.94 [1.45–2.59]	7.06E-06	I
rs10053849	5	50781062	A/G	0.395	0.252	0.264	1.93 [1.44–2.57]	8.65E-06	-
rs11960393	5	50781964	G/A	0.395	0.252	0.264	1.93 [1.44–2.57]	8.81E-06	I
rs1559280	5	50780387	A/C	0.395	0.252	0.264	1.92 [1.44–2.57]	9.03E-06	I
rs6899166	5	50781314	C/T	0.395	0.252	0.264	1.92 [1.44–2.57]	9.06E-06	I
rs1559279	5	50781110	G/T	0.394	0.252	0.264	1.92 [1.44–2.56]	9.52E-06	I
