# Supplementary material for: Genome-wide Association Study and Meta-Analysis Identify ISL1 as Genome-wide Significant Susceptibility Gene for Bladder Exstrophy
Source: PLoS Genet. 2015 Mar 12;11(3):e1005024. doi: 10.1371/journal.pgen.1005024 (PMC4357422; doi:10.1371/journal.pgen.1005024)
Supplement: S2 Table — Relative risks (RRs) are given with the risk allele set as baseline. Chr, chromosome; RAF, risk allele frequency. (PDF) [file pgen.1005024.s002.pdf]

Supplementary Tables 2. Meta-analysis results for classic bladder exstrophy

				GWAS1- subgroup A						GWAS1 - subgroup B						GWAS2						Meta-analysis				
**SNP**	**Chr**	**Position**	**Risk/ other allel**	**RAF cases**	**RAF controls**	**RAF combined**	**RR [95% CI]**	***P* value**	**Imputed**	**RAF cases**	**RAF controls**	**RAF combined**	**RR [95% CI]**	***P* value**	**Imputed**	**RAF cases**	**RAF controls**	**RAF combined**	**RR [95% CI]**	***P* value**	**Imputed**	**RAF cases**	**RAF controls**	**RAF combined**	**RR [95% CI]**	***P* value**
rs9291768	5	50717793	T/C	0.628	0.413	0.444	2.50 [1.47-4.26]	7.62E-04	I	0.479	0.399	0.413	1.38 [0.90-2.10]	1.38E-01	I	0.605	0.364	0.384	2.60 [1.95-3.47]	8.89E-11	I	0.576	0.377	0.399	2.18 [1.75-2.71]	2.13E-12
rs6874700	5	50701750	A/T	0.628	0.412	0.443	2.53 [1.49-4.31]	6.36E-04	I	0.473	0.401	0.414	1.33 [0.87-2.02]	1.83E-01	I	0.605	0.362	0.383	2.62 [1.96-3.49]	6.27E-11	I	0.575	0.376	0.398	2.17 [1.75-2.70]	2.54E-12
rs10454819	5	50718216	A/G	0.628	0.403	0.435	2.74 [1.58-4.73]	3.06E-04	I	0.470	0.397	0.409	1.34 [0.87-2.04]	1.82E-01	I	0.585	0.360	0.379	2.49 [1.86-3.32]	6.95E-10	I	0.563	0.372	0.393	2.14 [1.72-2.66]	1.01E-11
rs6449609	5	50696967	A/T	0.634	0.438	0.466	2.28 [1.35-3.87]	2.19E-03	I	0.484	0.418	0.429	1.31 [0.87-1.98]	2.00E-01	I	0.607	0.380	0.399	2.51 [1.88-3.36]	5.02E-10	I	0.58	0.395	0.415	2.07 [1.66-2.56]	5.18E-11
rs4865663	5	50702964	T/C	0.622	0.428	0.456	2.23 [1.32-3.77]	2.73E-03	I	0.469	0.404	0.415	1.29 [0.86-1.95]	2.21E-01	I	0.600	0.369	0.389	2.52 [1.89-3.37]	3.30E-10	I	0.57	0.384	0.404	2.06 [1.66-2.55]	5.36E-11
rs10039350	5	50737297	C/T	0.628	0.434	0.461	2.21 [1.32-3.72]	2.74E-03	I	0.481	0.410	0.422	1.32 [0.87-2.00]	1.86E-01	I	0.604	0.377	0.396	2.48 [1.86-3.30]	7.45E-10	I	0.576	0.391	0.411	2.05 [1.65-2.54]	6.70E-11
rs7714459	5	50731536	C/T	0.628	0.434	0.461	2.21 [1.32-3.72]	2.76E-03	I	0.481	0.410	0.422	1.32 [0.87-2.00]	1.87E-01	I	0.603	0.377	0.396	2.47 [1.85-3.30]	8.46E-10	I	0.576	0.391	0.411	2.05 [1.65-2.54]	7.53E-11
rs6895297	5	50695877	A/G	0.635	0.436	0.464	2.30 [1.36-3.90]	1.93E-03	I	0.473	0.414	0.424	1.26 [0.84-1.90]	2.63E-01	-	0.605	0.375	0.395	2.50 [1.87-3.34]	4.91E-10	-	0.576	0.39	0.411	2.04 [1.65-2.53]	8.08E-11
rs13356912	5	50738088	G/A	0.628	0.436	0.463	2.20 [1.31-3.70]	3.04E-03	I	0.481	0.410	0.422	1.32 [0.87-2.00]	1.87E-01	I	0.604	0.378	0.397	2.47 [1.85-3.30]	8.42E-10	I	0.576	0.392	0.412	2.04 [1.65-2.54]	8.14E-11
rs10041557	5	50734335	G/C	0.628	0.436	0.463	2.20 [1.31-3.71]	3.02E-03	I	0.481	0.410	0.422	1.32 [0.87-2.00]	1.86E-01	I	0.604	0.378	0.397	2.47 [1.85-3.30]	8.56E-10	I	0.576	0.392	0.412	2.05 [1.65-2.54]	8.17E-11
rs10939960	5	50738447	T/C	0.628	0.437	0.464	2.19 [1.30-3.68]	3.24E-03	I	0.482	0.410	0.422	1.32 [0.87-2.00]	1.85E-01	I	0.605	0.379	0.398	2.47 [1.85-3.30]	8.15E-10	I	0.577	0.393	0.413	2.04 [1.65-2.54]	8.30E-11
rs4865666	5	50737629	T/A	0.628	0.437	0.464	2.19 [1.30-3.68]	3.24E-03	I	0.482	0.410	0.422	1.32 [0.87-2.00]	1.85E-01	I	0.605	0.379	0.398	2.47 [1.85-3.30]	8.19E-10	I	0.577	0.393	0.413	2.04 [1.65-2.54]	8.34E-11
rs7717071	5	50739735	T/C	0.624	0.435	0.462	2.17 [1.29-3.66]	3.55E-03	I	0.481	0.409	0.421	1.33 [0.88-2.02]	1.78E-01	I	0.604	0.378	0.398	2.47 [1.85-3.30]	8.38E-10	I	0.576	0.392	0.412	2.05 [1.65-2.54]	8.35E-11
rs10040678	5	50737159	A/G	0.628	0.436	0.463	2.20 [1.31-3.71]	3.01E-03	I	0.481	0.410	0.422	1.32 [0.87-2.00]	1.88E-01	I	0.604	0.378	0.397	2.47 [1.85-3.29]	8.69E-10	I	0.576	0.392	0.412	2.04 [1.65-2.54]	8.35E-11
rs10043658	5	50733422	G/A	0.628	0.436	0.463	2.20 [1.31-3.70]	3.05E-03	I	0.482	0.410	0.422	1.32 [0.87-2.00]	1.85E-01	-	0.604	0.378	0.398	2.47 [1.85-3.29]	8.85E-10	I	0.577	0.392	0.412	2.04 [1.65-2.54]	8.38E-11
rs10939961	5	50738478	A/C	0.628	0.436	0.464	2.19 [1.30-3.69]	3.17E-03	-	0.481	0.410	0.422	1.32 [0.87-2.00]	1.87E-01	I	0.604	0.378	0.397	2.47 [1.85-3.30]	8.40E-10	I	0.576	0.392	0.412	2.04 [1.65-2.54]	8.42E-11
rs11951998	5	50736314	C/T	0.628	0.437	0.464	2.19 [1.30-3.68]	3.25E-03	I	0.482	0.410	0.422	1.32 [0.87-2.00]	1.85E-01	-	0.605	0.379	0.398	2.47 [1.85-3.30]	8.43E-10	-	0.577	0.392	0.412	2.04 [1.65-2.54]	8.52E-11
rs7733960	5	50735905	T/C	0.628	0.436	0.463	2.20 [1.31-3.70]	3.05E-03	I	0.481	0.410	0.422	1.32 [0.87-2.00]	1.88E-01	I	0.604	0.378	0.397	2.47 [1.85-3.29]	8.73E-10	I	0.576	0.392	0.412	2.04 [1.65-2.53]	8.52E-11
rs6865525	5	50734787	A/G	0.628	0.436	0.463	2.20 [1.31-3.71]	3.02E-03	I	0.481	0.410	0.422	1.32 [0.87-2.00]	1.88E-01	I	0.604	0.378	0.398	2.47 [1.85-3.29]	8.90E-10	I	0.576	0.392	0.412	2.04 [1.65-2.53]	8.56E-11
rs6891088	5	50734414	C/T	0.628	0.436	0.463	2.20 [1.31-3.70]	3.05E-03	I	0.481	0.410	0.422	1.32 [0.87-2.00]	1.88E-01	I	0.604	0.378	0.398	2.47 [1.85-3.29]	8.89E-10	I	0.576	0.392	0.412	2.04 [1.65-2.53]	8.67E-11
rs7713979	5	50714556	G/A	0.628	0.436	0.463	2.19 [1.30-3.69]	3.15E-03	I	0.476	0.411	0.422	1.29 [0.85-1.95]	2.26E-01	I	0.605	0.377	0.397	2.50 [1.87-3.35]	5.41E-10	I	0.576	0.391	0.412	2.04 [1.65-2.54]	9.03E-11
rs10454820	5	50718769	A/G	0.628	0.437	0.464	2.19 [1.30-3.68]	3.23E-03	I	0.480	0.411	0.423	1.31 [0.87-1.98]	2.00E-01	I	0.606	0.379	0.398	2.49 [1.86-3.33]	7.28E-10	I	0.577	0.393	0.413	2.04 [1.65-2.54]	9.04E-11
rs9291766	5	50715254	A/G	0.628	0.436	0.463	2.19 [1.30-3.69]	3.16E-03	I	0.476	0.411	0.422	1.29 [0.85-1.96]	2.24E-01	I	0.605	0.377	0.397	2.50 [1.87-3.34]	5.64E-10	I	0.576	0.392	0.412	2.04 [1.65-2.54]	9.17E-11
rs10939954	5	50724774	G/A	0.628	0.436	0.464	2.19 [1.30-3.69]	3.19E-03	I	0.481	0.410	0.422	1.32 [0.87-2.00]	1.91E-01	I	0.604	0.378	0.397	2.47 [1.85-3.30]	8.72E-10	I	0.576	0.392	0.412	2.04 [1.65-2.53]	9.19E-11
rs4865664	5	50722238	C/A	0.628	0.437	0.464	2.19 [1.30-3.68]	3.22E-03	I	0.482	0.410	0.422	1.32 [0.87-2.00]	1.85E-01	-	0.605	0.379	0.399	2.47 [1.85-3.30]	9.28E-10	-	0.577	0.393	0.413	2.04 [1.65-2.53]	9.21E-11
rs10066455	5	50721742	A/G	0.628	0.437	0.464	2.19 [1.30-3.69]	3.21E-03	I	0.481	0.411	0.423	1.32 [0.87-1.99]	1.93E-01	I	0.605	0.380	0.399	2.48 [1.86-3.31]	8.35E-10	I	0.577	0.393	0.413	2.04 [1.65-2.54]	9.28E-11
rs4865667	5	50748173	T/C	0.618	0.436	0.462	2.11 [1.25-3.55]	5.03E-03	I	0.482	0.406	0.419	1.35 [0.89-2.05]	1.58E-01	-	0.605	0.379	0.399	2.48 [1.86-3.32]	8.69E-10	-	0.575	0.392	0.412	2.05 [1.65-2.54]	9.45E-11
rs7721741	5	50741017	C/G	0.620	0.435	0.462	2.13 [1.26-3.58]	4.51E-03	I	0.482	0.407	0.420	1.35 [0.89-2.04]	1.63E-01	I	0.605	0.380	0.399	2.48 [1.85-3.31]	9.31E-10	I	0.575	0.393	0.413	2.05 [1.65-2.54]	9.53E-11
rs10066521	5	50721825	T/G	0.628	0.437	0.464	2.19 [1.30-3.68]	3.22E-03	I	0.480	0.410	0.422	1.32 [0.87-1.99]	1.93E-01	I	0.604	0.378	0.398	2.47 [1.85-3.30]	8.81E-10	I	0.576	0.392	0.412	2.04 [1.64-2.53]	9.58E-11
rs28664909	5	50706499	A/G	0.628	0.436	0.464	2.19 [1.30-3.69]	3.16E-03	I	0.474	0.413	0.423	1.27 [0.84-1.92]	2.54E-01	I	0.605	0.376	0.396	2.51 [1.88-3.35]	4.40E-10	I	0.575	0.391	0.411	2.04 [1.64-2.53]	9.90E-11
rs6449622	5	50709369	G/C	0.628	0.436	0.464	2.19 [1.30-3.69]	3.17E-03	I	0.475	0.413	0.423	1.28 [0.85-1.93]	2.45E-01	I	0.606	0.377	0.397	2.51 [1.88-3.36]	4.83E-10	I	0.576	0.392	0.412	2.04 [1.64-2.53]	1.00E-10
rs7714281	5	50731380	A/T	0.628	0.437	0.464	2.19 [1.30-3.68]	3.25E-03	I	0.482	0.410	0.422	1.32 [0.87-2.00]	1.86E-01	I	0.605	0.380	0.399	2.46 [1.85-3.29]	1.01E-09	I	0.577	0.393	0.413	2.04 [1.64-2.53]	1.00E-10
rs62368261	5	50722381	A/G	0.628	0.437	0.464	2.19 [1.30-3.68]	3.29E-03	I	0.481	0.411	0.423	1.32 [0.87-1.99]	1.95E-01	I	0.604	0.379	0.398	2.47 [1.85-3.30]	9.07E-10	I	0.576	0.393	0.413	2.04 [1.64-2.53]	1.01E-10
rs4865658	5	50680233	A/G	0.628	0.434	0.462	2.23 [1.32-3.77]	2.68E-03	I	0.473	0.416	0.425	1.26 [0.83-1.90]	2.74E-01	I	0.604	0.376	0.395	2.52 [1.89-3.37]	4.20E-10	I	0.574	0.391	0.411	2.04 [1.64-2.53]	1.02E-10
rs6449623	5	50712332	G/C	0.628	0.436	0.464	2.20 [1.30-3.70]	3.11E-03	I	0.476	0.412	0.423	1.29 [0.85-1.95]	2.32E-01	I	0.606	0.378	0.398	2.50 [1.87-3.34]	5.94E-10	I	0.576	0.392	0.412	2.04 [1.64-2.53]	1.03E-10
rs6861333	5	50703933	G/C	0.628	0.436	0.464	2.19 [1.30-3.69]	3.17E-03	I	0.473	0.413	0.424	1.27 [0.84-1.91]	2.62E-01	I	0.607	0.377	0.397	2.52 [1.88-3.36]	4.39E-10	I	0.576	0.392	0.412	2.03 [1.64-2.52]	1.09E-10
rs10939963	5	50747502	C/A	0.620	0.440	0.466	2.10 [1.25-3.53]	5.34E-03	I	0.489	0.408	0.421	1.38 [0.91-2.10]	1.30E-01	I	0.609	0.384	0.404	2.44 [1.83-3.27]	1.56E-09	I	0.58	0.396	0.416	2.04 [1.64-2.54]	1.09E-10
rs6449612	5	50701102	T/C	0.628	0.436	0.464	2.19 [1.30-3.69]	3.17E-03	-	0.473	0.414	0.424	1.26 [0.84-1.91]	2.67E-01	I	0.605	0.376	0.395	2.51 [1.88-3.35]	4.40E-10	I	0.575	0.391	0.411	2.03 [1.64-2.52]	1.12E-10
rs973860	5	50701139	T/C	0.628	0.436	0.463	2.19 [1.30-3.69]	3.18E-03	I	0.473	0.414	0.424	1.26 [0.84-1.91]	2.67E-01	I	0.605	0.376	0.395	2.51 [1.88-3.35]	4.40E-10	I	0.575	0.391	0.411	2.03 [1.64-2.52]	1.12E-10
rs1158641	5	50703285	A/C	0.628	0.436	0.464	2.19 [1.30-3.69]	3.17E-03	I	0.473	0.414	0.424	1.26 [0.84-1.91]	2.64E-01	I	0.607	0.377	0.397	2.51 [1.88-3.36]	4.50E-10	I	0.576	0.392	0.412	2.03 [1.64-2.52]	1.12E-10
rs991216	5	50688533	G/C	0.616	0.434	0.460	2.21 [1.31-3.73]	3.05E-03	I	0.473	0.415	0.425	1.26 [0.83-1.90]	2.73E-01	I	0.604	0.374	0.394	2.51 [1.88-3.36]	4.27E-10	I	0.572	0.389	0.409	2.03 [1.64-2.52]	1.14E-10
rs2303751	5	50685505	G/A	0.628	0.435	0.462	2.22 [1.31-3.75]	2.85E-03	I	0.473	0.416	0.425	1.26 [0.83-1.90]	2.74E-01	I	0.605	0.375	0.395	2.50 [1.88-3.34]	4.67E-10	I	0.575	0.39	0.41	2.03 [1.64-2.52]	1.14E-10
rs991217	5	50688801	A/G	0.628	0.435	0.462	2.22 [1.31-3.74]	2.89E-03	I	0.473	0.416	0.425	1.26 [0.83-1.90]	2.74E-01	I	0.605	0.375	0.395	2.50 [1.88-3.34]	4.65E-10	I	0.575	0.39	0.411	2.03 [1.64-2.52]	1.15E-10
rs6449610	5	50698339	C/T	0.628	0.436	0.463	2.20 [1.31-3.71]	3.08E-03	I	0.473	0.415	0.425	1.26 [0.84-1.90]	2.70E-01	I	0.605	0.375	0.395	2.51 [1.88-3.35]	4.59E-10	I	0.575	0.391	0.411	2.03 [1.64-2.52]	1.17E-10
rs7705789	5	50737991	A/G	0.624	0.434	0.461	2.19 [1.30-3.68]	3.28E-03	I	0.480	0.409	0.421	1.32 [0.87-2.00]	1.87E-01	I	0.597	0.375	0.394	2.46 [1.84-3.28]	1.22E-09	I	0.572	0.389	0.409	2.04 [1.64-2.53]	1.19E-10
rs10076515	5	50702024	A/G	0.628	0.436	0.464	2.19 [1.30-3.69]	3.18E-03	I	0.473	0.414	0.424	1.26 [0.84-1.90]	2.68E-01	I	0.606	0.377	0.397	2.51 [1.88-3.35]	4.70E-10	I	0.576	0.392	0.412	2.03 [1.64-2.52]	1.20E-10
rs10040820	5	50694579	G/A	0.628	0.435	0.463	2.21 [1.31-3.72]	2.99E-03	I	0.473	0.416	0.425	1.26 [0.83-1.90]	2.76E-01	-	0.605	0.375	0.395	2.50 [1.88-3.34]	4.71E-10	I	0.575	0.391	0.411	2.03 [1.64-2.52]	1.22E-10
rs10041392	5	50700597	G/A	0.628	0.436	0.463	2.19 [1.30-3.69]	3.16E-03	I	0.473	0.415	0.424	1.26 [0.84-1.90]	2.70E-01	I	0.606	0.377	0.396	2.51 [1.88-3.35]	4.79E-10	I	0.575	0.392	0.412	2.03 [1.64-2.52]	1.24E-10
rs6869470	5	50663015	T/C	0.628	0.433	0.460	2.30 [1.35-3.90]	2.08E-03	I	0.472	0.414	0.424	1.26 [0.84-1.90]	2.71E-01	I	0.597	0.372	0.391	2.47 [1.85-3.30]	7.49E-10	I	0.57	0.388	0.407	2.03 [1.64-2.52]	1.24E-10
rs10039723	5	50739152	C/A	0.626	0.435	0.462	2.19 [1.30-3.68]	3.29E-03	I	0.482	0.412	0.424	1.31 [0.87-1.99]	1.99E-01	-	0.605	0.381	0.400	2.46 [1.84-3.29]	1.15E-09	-	0.577	0.394	0.414	2.03 [1.64-2.53]	1.27E-10
rs1017	5	50690095	T/A	0.628	0.435	0.463	2.22 [1.31-3.74]	2.90E-03	I	0.473	0.416	0.426	1.26 [0.83-1.89]	2.77E-01	I	0.606	0.376	0.396	2.50 [1.87-3.34]	5.15E-10	I	0.575	0.391	0.411	2.03 [1.63-2.51]	1.30E-10
rs4865662	5	50699552	G/A	0.628	0.436	0.464	2.20 [1.30-3.70]	3.13E-03	I	0.473	0.415	0.425	1.26 [0.84-1.90]	2.70E-01	I	0.607	0.378	0.397	2.50 [1.87-3.35]	5.17E-10	I	0.576	0.392	0.412	2.03 [1.63-2.52]	1.31E-10
rs6861877	5	50691723	C/G	0.628	0.435	0.463	2.21 [1.31-3.73]	2.95E-03	I	0.473	0.416	0.426	1.26 [0.83-1.89]	2.78E-01	I	0.606	0.376	0.396	2.50 [1.87-3.34]	5.18E-10	I	0.575	0.391	0.411	2.03 [1.63-2.51]	1.33E-10
rs7709134	5	50693176	A/G	0.628	0.436	0.463	2.21 [1.31-3.73]	2.98E-03	I	0.473	0.416	0.426	1.26 [0.83-1.89]	2.76E-01	I	0.606	0.377	0.397	2.50 [1.87-3.34]	5.49E-10	I	0.576	0.392	0.412	2.03 [1.63-2.51]	1.39E-10
rs2288468	5	50683655	C/T	0.628	0.435	0.462	2.22 [1.32-3.75]	2.83E-03	I	0.473	0.416	0.425	1.26 [0.83-1.90]	2.76E-01	-	0.605	0.377	0.396	2.48 [1.86-3.31]	6.35E-10	-	0.575	0.392	0.412	2.02 [1.63-2.51]	1.45E-10
rs2115322	5	50666618	G/A	0.628	0.434	0.462	2.23 [1.32-3.77]	2.66E-03	-	0.473	0.415	0.425	1.26 [0.84-1.90]	2.69E-01	I	0.602	0.376	0.396	2.49 [1.86-3.33]	6.89E-10	I	0.573	0.391	0.411	2.03 [1.63-2.51]	1.46E-10
rs6859394	5	50686582	C/T	0.628	0.435	0.463	2.22 [1.32-3.75]	2.84E-03	I	0.473	0.416	0.426	1.26 [0.83-1.90]	2.75E-01	I	0.606	0.378	0.398	2.50 [1.87-3.33]	6.26E-10	I	0.575	0.393	0.413	2.03 [1.63-2.51]	1.48E-10
rs4865512	5	50661601	G/A	0.628	0.435	0.462	2.24 [1.32-3.79]	2.64E-03	I	0.472	0.414	0.424	1.26 [0.83-1.90]	2.74E-01	I	0.601	0.376	0.395	2.49 [1.86-3.32]	7.22E-10	I	0.572	0.391	0.41	2.02 [1.63-2.51]	1.53E-10
rs6449608	5	50696922	A/T	0.624	0.425	0.454	2.30 [1.35-3.90]	2.03E-03	I	0.451	0.394	0.403	1.28 [0.84-1.94]	2.58E-01	I	0.571	0.359	0.378	2.39 [1.80-3.19]	2.78E-09	I	0.55	0.375	0.394	2.01 [1.62-2.50]	2.56E-10
rs9291770	5	50719408	G/T	0.615	0.430	0.457	2.14 [1.27-3.60]	4.03E-03	I	0.471	0.404	0.416	1.31 [0.86-1.99]	2.09E-01	I	0.592	0.375	0.393	2.42 [1.81-3.24]	2.35E-09	I	0.565	0.388	0.407	2.01 [1.62-2.50]	2.78E-10
rs4865656	5	50659788	A/G	0.628	0.432	0.460	2.30 [1.35-3.91]	2.07E-03	-	0.471	0.414	0.424	1.26 [0.83-1.90]	2.74E-01	I	0.586	0.371	0.389	2.38 [1.78-3.16]	3.17E-09	-	0.565	0.387	0.406	1.99 [1.60-2.46]	3.77E-10
rs4343805	5	50648159	T/C	0.617	0.436	0.461	2.34 [1.34-4.09]	2.90E-03	I	0.496	0.428	0.440	1.38 [0.89-2.12]	1.48E-01	I	0.581	0.386	0.403	2.37 [1.76-3.21]	2.05E-08	I	0.566	0.4	0.418	2.04 [1.63-2.56]	6.93E-10
rs10939913	5	50592825	C/G	0.442	0.270	0.295	2.75 [1.55-4.89]	5.72E-04	I	0.325	0.280	0.287	1.26 [0.79-2.00]	3.26E-01	I	0.427	0.249	0.265	2.26 [1.69-3.02]	4.33E-08	I	0.403	0.257	0.273	2.02 [1.61-2.54]	1.11E-09
rs2405154	5	50625038	G/A	0.651	0.459	0.486	2.38 [1.38-4.12]	1.87E-03	I	0.509	0.451	0.461	1.30 [0.86-1.98]	2.13E-01	I	0.623	0.419	0.437	2.26 [1.69-3.02]	3.62E-08	I	0.599	0.43	0.449	1.96 [1.58-2.44]	1.50E-09
rs7349819	5	50590195	T/G	0.651	0.466	0.493	2.39 [1.38-4.15]	1.87E-03	I	0.512	0.454	0.464	1.30 [0.85-1.98]	2.25E-01	I	0.627	0.423	0.440	2.27 [1.69-3.04]	3.65E-08	I	0.601	0.434	0.453	1.96 [1.58-2.45]	1.68E-09
rs6449585	5	50632997	G/A	0.651	0.461	0.488	2.36 [1.36-4.07]	2.09E-03	I	0.509	0.451	0.461	1.30 [0.86-1.98]	2.13E-01	I	0.623	0.419	0.437	2.25 [1.69-3.01]	3.78E-08	I	0.598	0.431	0.449	1.96 [1.57-2.43]	1.69E-09
rs6414789	5	50634836	A/T	0.651	0.461	0.488	2.35 [1.36-4.07]	2.11E-03	I	0.509	0.451	0.461	1.30 [0.86-1.98]	2.14E-01	I	0.623	0.420	0.437	2.25 [1.69-3.01]	3.78E-08	I	0.598	0.431	0.449	1.96 [1.57-2.43]	1.71E-09
rs6872771	5	50653635	C/T	0.651	0.465	0.491	2.30 [1.33-3.97]	2.95E-03	I	0.513	0.453	0.463	1.32 [0.87-2.00]	2.00E-01	I	0.623	0.421	0.438	2.28 [1.70-3.05]	3.36E-08	I	0.6	0.432	0.451	1.96 [1.57-2.44]	1.82E-09
rs4481304	5	50759415	A/G	0.631	0.483	0.505	1.94 [1.16-3.25]	1.17E-02	I	0.477	0.441	0.447	1.17 [0.77-1.78]	4.57E-01	I	0.648	0.420	0.440	2.54 [1.89-3.42]	7.02E-10	I	0.599	0.433	0.451	1.96 [1.57-2.44]	1.84E-09
rs2195406	5	50644455	G/C	0.651	0.462	0.489	2.38 [1.37-4.13]	2.16E-03	I	0.511	0.452	0.462	1.31 [0.86-1.99]	2.05E-01	I	0.618	0.418	0.435	2.26 [1.69-3.02]	4.39E-08	I	0.597	0.43	0.448	1.96 [1.57-2.44]	1.90E-09
rs6867206	5	50652694	C/T	0.651	0.463	0.490	2.35 [1.35-4.08]	2.46E-03	-	0.513	0.453	0.463	1.32 [0.87-2.00]	1.99E-01	I	0.618	0.418	0.435	2.26 [1.69-3.02]	4.30E-08	I	0.597	0.431	0.449	1.96 [1.57-2.44]	1.94E-09
rs7702670	5	50636650	T/C	0.651	0.462	0.489	2.35 [1.36-4.06]	2.16E-03	I	0.509	0.451	0.461	1.30 [0.86-1.98]	2.15E-01	-	0.623	0.421	0.438	2.25 [1.68-3.01]	4.28E-08	-	0.599	0.432	0.45	1.95 [1.57-2.43]	1.95E-09
rs6881501	5	50652823	A/G	0.651	0.464	0.490	2.35 [1.35-4.08]	2.46E-03	I	0.513	0.453	0.463	1.32 [0.87-2.00]	2.00E-01	I	0.619	0.419	0.436	2.26 [1.69-3.03]	4.28E-08	I	0.597	0.431	0.449	1.96 [1.57-2.44]	1.95E-09
rs9687742	5	50647350	C/T	0.650	0.459	0.486	2.40 [1.38-4.16]	1.94E-03	I	0.511	0.451	0.461	1.32 [0.87-2.01]	1.98E-01	I	0.615	0.418	0.435	2.25 [1.68-3.01]	5.57E-08	I	0.595	0.429	0.447	1.96 [1.57-2.44]	1.97E-09
rs7709389	5	50656032	C/G	0.648	0.463	0.490	2.34 [1.34-4.07]	2.72E-03	I	0.513	0.453	0.463	1.32 [0.87-2.00]	1.98E-01	I	0.618	0.418	0.435	2.26 [1.69-3.03]	4.23E-08	I	0.596	0.431	0.449	1.96 [1.57-2.44]	2.08E-09

				GWAS1- subgroup A						GWAS1 - subgroup B						GWAS2						Meta-analysis				
SNP	Chr	Position	Risk/ other allel	RAF cases	RAF controls	RAF combined	RR [95% CI]	*P* value	Imputed	RAF cases	RAF controls	RAF combined	RR [95% CI]	*P* value	Imputed	RAF cases	RAF controls	RAF combined	RR [95% CI]	*P* value	Imputed	RAF cases	RAF controls	RAF combined	RR [95% CI]	*P* value
rs2217316	5	50656853	A/G	0.646	0.463	0.489	2.33 [1.33-4.06]	2.90E-03	I	0.513	0.453	0.463	1.32 [0.87-2.00]	1.98E-01	I	0.618	0.418	0.435	2.26 [1.69-3.03]	4.24E-08	I	0.596	0.431	0.449	1.96 [1.57-2.44]	2.18E-09
rs9687615	5	50647257	C/A	0.651	0.455	0.483	2.46 [1.40-4.30]	1.65E-03	I	0.507	0.450	0.460	1.31 [0.86-1.99]	2.16E-01	I	0.613	0.417	0.434	2.23 [1.66-2.99]	7.43E-08	I	0.593	0.428	0.446	1.96 [1.57-2.44]	2.59E-09
rs6414788	5	50634777	C/T	0.640	0.456	0.482	2.27 [1.32-3.89]	2.94E-03	I	0.503	0.450	0.459	1.28 [0.84-1.95]	2.47E-01	I	0.621	0.418	0.436	2.26 [1.69-3.02]	3.80E-08	I	0.594	0.429	0.447	1.94 [1.56-2.41]	2.85E-09
rs10037522	5	50635643	C/A	0.651	0.461	0.488	2.35 [1.36-4.06]	2.12E-03	I	0.500	0.451	0.460	1.26 [0.83-1.92]	2.74E-01	-	0.623	0.421	0.438	2.24 [1.68-3.00]	4.64E-08	-	0.596	0.432	0.45	1.93 [1.56-2.41]	3.20E-09
rs1501985	5	50595433	C/T	0.647	0.462	0.488	2.36 [1.37-4.06]	2.08E-03	I	0.509	0.450	0.460	1.31 [0.86-1.99]	2.13E-01	I	0.616	0.419	0.435	2.20 [1.65-2.94]	9.08E-08	I	0.594	0.43	0.448	1.93 [1.55-2.41]	3.35E-09
rs1559278	5	50794221	C/T	0.581	0.400	0.425	2.07 [1.23-3.50]	6.33E-03	-	0.445	0.356	0.371	1.50 [0.97-2.30]	6.58E-02	-	0.541	0.364	0.379	2.08 [1.57-2.76]	4.09E-07	-	0.524	0.368	0.385	1.91 [1.54-2.37]	3.66E-09
rs1423618	5	50800179	C/T	0.582	0.399	0.425	2.08 [1.23-3.51]	6.08E-03	I	0.446	0.354	0.370	1.49 [0.97-2.28]	6.72E-02	I	0.541	0.363	0.378	2.08 [1.57-2.77]	4.02E-07	I	0.524	0.367	0.384	1.91 [1.54-2.37]	3.70E-09
rs10066412	5	50600177	T/A	0.647	0.461	0.488	2.36 [1.37-4.07]	2.05E-03	I	0.509	0.450	0.460	1.31 [0.86-1.99]	2.12E-01	I	0.614	0.418	0.435	2.19 [1.64-2.93]	1.05E-07	I	0.593	0.43	0.448	1.93 [1.55-2.40]	3.71E-09
rs10067551	5	50640597	C/A	0.652	0.466	0.493	2.32 [1.34-4.03]	2.75E-03	I	0.536	0.470	0.482	1.39 [0.90-2.14]	1.41E-01	I	0.623	0.438	0.454	2.14 [1.60-2.86]	3.36E-07	I	0.606	0.447	0.465	1.93 [1.55-2.42]	5.46E-09
rs10077073	5	50631992	A/G	0.431	0.229	0.258	3.08 [1.72-5.51]	1.57E-04	I	0.267	0.217	0.226	1.35 [0.83-2.20]	2.31E-01	I	0.357	0.214	0.226	2.08 [1.54-2.81]	1.74E-06	I	0.349	0.217	0.231	2.01 [1.59-2.54]	5.79E-09
rs6449543	5	50590024	C/T	0.446	0.286	0.309	2.48 [1.41-4.37]	1.63E-03	I	0.331	0.291	0.297	1.22 [0.78-1.93]	3.84E-01	I	0.439	0.264	0.279	2.20 [1.65-2.94]	9.25E-08	I	0.412	0.271	0.287	1.95 [1.55-2.44]	6.11E-09
rs10067157	5	50645956	G/A	0.426	0.223	0.252	3.18 [1.76-5.74]	1.28E-04	I	0.261	0.213	0.222	1.32 [0.81-2.16]	2.60E-01	I	0.350	0.209	0.221	2.06 [1.52-2.78]	2.56E-06	I	0.342	0.212	0.226	1.99 [1.57-2.52]	9.48E-09
rs10045426	5	50619895	A/C	0.430	0.229	0.258	3.08 [1.72-5.50]	1.54E-04	I	0.260	0.215	0.223	1.31 [0.80-2.14]	2.86E-01	I	0.355	0.213	0.225	2.07 [1.53-2.80]	2.11E-06	I	0.346	0.216	0.23	1.99 [1.57-2.52]	9.49E-09
rs72499502	5	50603530	A/G	0.426	0.225	0.254	3.15 [1.74-5.70]	1.45E-04	I	0.255	0.209	0.217	1.31 [0.80-2.15]	2.78E-01	I	0.349	0.207	0.219	2.06 [1.53-2.78]	2.34E-06	I	0.34	0.21	0.224	1.99 [1.57-2.52]	1.01E-08
rs16878735	5	50598614	T/A	0.426	0.227	0.255	3.06 [1.70-5.49]	1.80E-04	I	0.253	0.209	0.216	1.32 [0.80-2.18]	2.80E-01	I	0.348	0.208	0.220	2.08 [1.54-2.82]	2.32E-06	I	0.339	0.211	0.225	2.00 [1.58-2.54]	1.03E-08
rs10040324	5	50632741	A/C	0.430	0.228	0.257	3.08 [1.72-5.51]	1.47E-04	I	0.263	0.217	0.225	1.31 [0.81-2.14]	2.73E-01	I	0.354	0.213	0.225	2.06 [1.52-2.78]	2.62E-06	I	0.345	0.216	0.23	1.98 [1.57-2.50]	1.08E-08
rs142059785	5	50602256	G/C	0.426	0.227	0.255	3.14 [1.73-5.68]	1.56E-04	I	0.255	0.209	0.217	1.31 [0.80-2.15]	2.79E-01	I	0.349	0.207	0.219	2.06 [1.52-2.78]	2.40E-06	I	0.34	0.21	0.224	1.99 [1.57-2.51]	1.09E-08
rs963195	5	50618929	G/C	0.430	0.229	0.258	3.07 [1.72-5.50]	1.57E-04	I	0.259	0.214	0.222	1.31 [0.80-2.14]	2.87E-01	I	0.354	0.212	0.224	2.06 [1.53-2.78]	2.43E-06	I	0.344	0.215	0.229	1.98 [1.57-2.51]	1.10E-08
rs7700319	5	50598539	C/T	0.442	0.282	0.305	2.46 [1.40-4.33]	1.80E-03	I	0.327	0.286	0.293	1.24 [0.78-1.96]	3.59E-01	I	0.427	0.259	0.274	2.16 [1.62-2.89]	2.12E-07	I	0.404	0.267	0.282	1.93 [1.54-2.42]	1.11E-08
rs10039627	5	50632050	T/C	0.430	0.228	0.257	3.08 [1.72-5.51]	1.47E-04	I	0.263	0.217	0.225	1.31 [0.81-2.14]	2.73E-01	I	0.353	0.213	0.225	2.06 [1.52-2.78]	2.69E-06	I	0.345	0.216	0.23	1.98 [1.57-2.50]	1.11E-08
rs10755219	5	50601746	C/T	0.427	0.279	0.300	2.49 [1.39-4.45]	2.13E-03	I	0.318	0.287	0.292	1.17 [0.73-1.86]	5.11E-01	I	0.429	0.254	0.269	2.25 [1.68-3.02]	7.34E-08	I	0.399	0.263	0.278	1.95 [1.55-2.45]	1.12E-08
rs1501987	5	50591998	T/C	0.442	0.282	0.304	2.47 [1.40-4.34]	1.76E-03	I	0.325	0.286	0.292	1.23 [0.78-1.95]	3.79E-01	I	0.427	0.259	0.273	2.17 [1.62-2.90]	1.96E-07	I	0.403	0.267	0.281	1.93 [1.54-2.42]	1.14E-08
rs9291746	5	50622094	T/C	0.430	0.228	0.257	3.08 [1.72-5.51]	1.48E-04	I	0.261	0.216	0.224	1.31 [0.80-2.14]	2.80E-01	I	0.353	0.212	0.224	2.05 [1.52-2.78]	2.70E-06	I	0.345	0.215	0.229	1.98 [1.57-2.50]	1.15E-08
rs2217314	5	50622582	C/T	0.430	0.228	0.257	3.08 [1.72-5.51]	1.47E-04	-	0.262	0.217	0.224	1.31 [0.80-2.14]	2.78E-01	I	0.353	0.213	0.225	2.05 [1.52-2.77]	2.76E-06	I	0.345	0.216	0.23	1.98 [1.57-2.50]	1.16E-08
rs10038557	5	50620779	C/T	0.430	0.228	0.257	3.08 [1.72-5.50]	1.52E-04	I	0.262	0.216	0.224	1.32 [0.81-2.15]	2.74E-01	I	0.354	0.213	0.225	2.05 [1.52-2.76]	3.00E-06	I	0.345	0.216	0.23	1.98 [1.56-2.50]	1.22E-08
rs11745678	5	50609478	G/C	0.430	0.230	0.258	3.06 [1.71-5.47]	1.66E-04	I	0.255	0.213	0.220	1.29 [0.79-2.12]	3.06E-01	I	0.353	0.211	0.223	2.06 [1.53-2.78]	2.41E-06	I	0.343	0.214	0.228	1.98 [1.56-2.50]	1.25E-08
rs10038485	5	50596639	T/A	0.430	0.230	0.258	3.06 [1.71-5.47]	1.68E-04	I	0.255	0.213	0.220	1.29 [0.79-2.12]	3.09E-01	I	0.354	0.211	0.224	2.06 [1.52-2.78]	2.45E-06	I	0.344	0.214	0.229	1.98 [1.56-2.50]	1.29E-08
rs10059280	5	50612871	A/G	0.430	0.231	0.260	3.05 [1.70-5.46]	1.77E-04	I	0.256	0.213	0.220	1.30 [0.79-2.13]	2.99E-01	I	0.353	0.211	0.223	2.06 [1.52-2.78]	2.49E-06	I	0.344	0.215	0.229	1.98 [1.56-2.50]	1.29E-08
rs143466268	5	50604309	T/C	0.430	0.230	0.258	3.06 [1.71-5.47]	1.67E-04	I	0.255	0.213	0.220	1.29 [0.79-2.11]	3.12E-01	I	0.354	0.211	0.224	2.06 [1.52-2.78]	2.49E-06	I	0.343	0.215	0.229	1.97 [1.56-2.50]	1.33E-08
rs6449541	5	50588359	T/C	0.430	0.229	0.258	3.07 [1.71-5.49]	1.65E-04	I	0.255	0.213	0.220	1.29 [0.79-2.12]	3.09E-01	I	0.354	0.212	0.224	2.05 [1.52-2.77]	2.60E-06	I	0.343	0.214	0.228	1.97 [1.56-2.50]	1.36E-08
rs72761990	5	50599662	A/G	0.430	0.232	0.260	3.05 [1.70-5.45]	1.79E-04	I	0.255	0.213	0.220	1.29 [0.79-2.12]	3.11E-01	I	0.354	0.212	0.224	2.06 [1.52-2.78]	2.51E-06	I	0.344	0.215	0.229	1.97 [1.56-2.50]	1.40E-08
rs7711781	5	50593076	T/C	0.430	0.231	0.260	3.05 [1.70-5.47]	1.77E-04	I	0.255	0.213	0.220	1.29 [0.79-2.12]	3.10E-01	I	0.354	0.212	0.224	2.06 [1.52-2.77]	2.55E-06	I	0.344	0.215	0.229	1.97 [1.56-2.50]	1.41E-08
rs10071749	5	50601535	C/T	0.430	0.232	0.260	3.05 [1.70-5.45]	1.79E-04	I	0.255	0.213	0.220	1.29 [0.79-2.12]	3.11E-01	I	0.354	0.212	0.224	2.06 [1.52-2.78]	2.54E-06	I	0.344	0.215	0.229	1.97 [1.56-2.49]	1.42E-08
rs963194	5	50618895	G/C	0.408	0.220	0.247	2.86 [1.60-5.11]	3.95E-04	I	0.238	0.206	0.211	1.22 [0.74-2.02]	4.37E-01	I	0.343	0.200	0.212	2.18 [1.60-2.97]	8.05E-07	I	0.329	0.204	0.217	2.00 [1.57-2.54]	1.48E-08
rs9291739	5	50608685	A/G	0.430	0.231	0.260	3.05 [1.70-5.46]	1.78E-04	I	0.255	0.213	0.220	1.28 [0.78-2.10]	3.21E-01	-	0.354	0.212	0.224	2.06 [1.52-2.78]	2.50E-06	I	0.343	0.215	0.229	1.97 [1.56-2.49]	1.48E-08
rs1423617	5	50801564	A/G	0.394	0.248	0.268	2.43 [1.34-4.42]	3.51E-03	I	0.301	0.223	0.236	1.57 [0.97-2.53]	6.49E-02	I	0.385	0.241	0.253	2.01 [1.50-2.71]	3.59E-06	I	0.364	0.239	0.252	1.95 [1.55-2.46]	1.52E-08
rs9291750	5	50636521	G/T	0.430	0.228	0.257	3.08 [1.72-5.51]	1.47E-04	I	0.264	0.217	0.225	1.31 [0.81-2.14]	2.71E-01	-	0.355	0.215	0.227	2.03 [1.50-2.74]	3.81E-06	-	0.346	0.217	0.231	1.97 [1.56-2.48]	1.53E-08
rs17398816	5	50595723	T/C	0.430	0.228	0.257	3.09 [1.73-5.53]	1.40E-04	-	0.255	0.212	0.220	1.30 [0.79-2.13]	3.02E-01	I	0.350	0.210	0.222	2.04 [1.51-2.75]	3.54E-06	I	0.341	0.213	0.227	1.97 [1.56-2.49]	1.56E-08
rs9291757	5	50653781	A/G	0.431	0.228	0.257	3.10 [1.73-5.56]	1.38E-04	I	0.261	0.217	0.225	1.29 [0.79-2.11]	3.01E-01	I	0.353	0.214	0.225	2.04 [1.51-2.76]	3.36E-06	I	0.345	0.216	0.23	1.97 [1.56-2.49]	1.58E-08
rs10055766	5	50654382	G/A	0.430	0.228	0.257	3.09 [1.73-5.52]	1.45E-04	I	0.260	0.217	0.224	1.29 [0.79-2.11]	3.01E-01	I	0.353	0.213	0.225	2.04 [1.51-2.76]	3.35E-06	I	0.344	0.216	0.23	1.97 [1.55-2.48]	1.61E-08
rs2897069	5	50654438	G/A	0.430	0.228	0.257	3.08 [1.72-5.52]	1.47E-04	-	0.260	0.217	0.224	1.29 [0.79-2.11]	3.01E-01	I	0.353	0.213	0.225	2.04 [1.51-2.76]	3.35E-06	I	0.344	0.216	0.23	1.96 [1.55-2.48]	1.62E-08
rs144781991	5	50649357	T/G	0.430	0.227	0.256	3.12 [1.74-5.58]	1.31E-04	I	0.260	0.217	0.224	1.29 [0.79-2.11]	3.04E-01	I	0.352	0.213	0.225	2.04 [1.51-2.76]	3.57E-06	I	0.344	0.216	0.23	1.97 [1.55-2.49]	1.64E-08
rs142461099	5	50602739	T/G	0.414	0.222	0.249	3.09 [1.70-5.60]	2.07E-04	I	0.249	0.208	0.215	1.28 [0.78-2.11]	3.28E-01	I	0.347	0.206	0.218	2.07 [1.53-2.79]	2.38E-06	I	0.335	0.208	0.222	1.98 [1.56-2.50]	1.66E-08
rs9291760	5	50655574	T/C	0.428	0.227	0.256	3.11 [1.74-5.59]	1.39E-04	I	0.259	0.217	0.224	1.29 [0.79-2.10]	3.12E-01	I	0.352	0.213	0.225	2.04 [1.51-2.75]	3.71E-06	I	0.343	0.216	0.23	1.96 [1.55-2.48]	1.87E-08
rs10052407	5	50591476	T/C	0.430	0.232	0.260	3.05 [1.70-5.46]	1.81E-04	I	0.255	0.213	0.220	1.28 [0.78-2.10]	3.21E-01	-	0.355	0.213	0.225	2.04 [1.51-2.75]	3.22E-06	-	0.344	0.216	0.23	1.96 [1.55-2.48]	1.90E-08
rs9291732	5	50584783	G/C	0.430	0.230	0.258	3.07 [1.71-5.50]	1.66E-04	I	0.255	0.214	0.221	1.29 [0.78-2.11]	3.20E-01	I	0.351	0.212	0.224	2.04 [1.51-2.76]	3.52E-06	I	0.342	0.215	0.229	1.96 [1.55-2.49]	1.92E-08
rs16875094	5	50734670	G/A	0.430	0.266	0.290	2.46 [1.42-4.29]	1.41E-03	I	0.300	0.241	0.251	1.36 [0.86-2.16]	1.88E-01	I	0.405	0.251	0.264	2.01 [1.51-2.68]	2.09E-06	-	0.382	0.252	0.266	1.89 [1.52-2.37]	2.07E-08
rs10939957	5	50727004	A/G	0.428	0.265	0.288	2.49 [1.43-4.34]	1.30E-03	I	0.300	0.239	0.249	1.39 [0.88-2.22]	1.62E-01	I	0.394	0.246	0.259	2.01 [1.50-2.69]	2.92E-06	I	0.376	0.248	0.262	1.91 [1.52-2.39]	2.09E-08
rs10063466	5	50647670	T/C	0.430	0.228	0.257	3.09 [1.73-5.52]	1.45E-04	I	0.261	0.217	0.224	1.30 [0.80-2.12]	2.91E-01	I	0.350	0.213	0.225	2.02 [1.50-2.73]	4.76E-06	I	0.343	0.216	0.23	1.96 [1.55-2.47]	2.11E-08
rs12521818	5	50583089	T/C	0.429	0.229	0.258	3.07 [1.71-5.50]	1.66E-04	I	0.256	0.215	0.222	1.29 [0.78-2.11]	3.20E-01	I	0.352	0.214	0.226	2.03 [1.50-2.75]	4.11E-06	I	0.342	0.216	0.23	1.96 [1.55-2.48]	2.22E-08
rs2033237	5	50596784	C/T	0.439	0.279	0.302	2.47 [1.40-4.35]	1.79E-03	I	0.327	0.287	0.293	1.24 [0.78-1.96]	3.61E-01	-	0.427	0.264	0.278	2.11 [1.58-2.82]	5.18E-07	-	0.403	0.27	0.284	1.90 [1.52-2.38]	2.42E-08
rs148490291	5	50604419	T/C	0.436	0.287	0.308	2.46 [1.38-4.38]	2.32E-03	I	0.334	0.298	0.304	1.21 [0.75-1.96]	4.32E-01	I	0.435	0.270	0.284	2.15 [1.60-2.88]	3.71E-07	I	0.408	0.277	0.291	1.92 [1.53-2.42]	2.65E-08
rs10075310	5	50649562	G/A	0.407	0.217	0.244	3.09 [1.70-5.61]	2.10E-04	I	0.243	0.208	0.214	1.29 [0.78-2.14]	3.25E-01	I	0.341	0.206	0.218	2.07 [1.52-2.81]	4.10E-06	I	0.329	0.208	0.221	1.98 [1.56-2.52]	2.67E-08
rs28557169	5	50720443	A/T	0.430	0.267	0.290	2.46 [1.41-4.27]	1.43E-03	I	0.300	0.242	0.252	1.36 [0.86-2.15]	1.92E-01	I	0.400	0.250	0.263	1.99 [1.49-2.66]	2.95E-06	I	0.38	0.251	0.265	1.88 [1.51-2.35]	2.95E-08
rs10036382	5	50612558	C/T	0.440	0.282	0.304	2.44 [1.38-4.30]	2.03E-03	I	0.316	0.287	0.292	1.17 [0.74-1.87]	5.02E-01	I	0.432	0.264	0.279	2.14 [1.60-2.87]	2.93E-07	-	0.403	0.271	0.285	1.90 [1.51-2.38]	2.96E-08
rs10471584	5	50759020	A/G	0.416	0.267	0.288	2.32 [1.32-4.06]	3.27E-03	I	0.302	0.237	0.248	1.42 [0.89-2.26]	1.40E-01	I	0.401	0.251	0.264	2.00 [1.50-2.68]	2.74E-06	I	0.378	0.251	0.265	1.89 [1.51-2.37]	3.00E-08
rs55867625	5	50599470	G/A	0.405	0.215	0.242	3.06 [1.69-5.55]	2.27E-04	I	0.236	0.204	0.209	1.24 [0.74-2.07]	4.21E-01	I	0.338	0.202	0.214	2.10 [1.54-2.87]	2.95E-06	I	0.325	0.205	0.218	1.99 [1.56-2.54]	3.05E-08
rs4141541	5	50584237	C/T	0.645	0.486	0.509	2.21 [1.27-3.84]	5.21E-03	I	0.510	0.468	0.475	1.22 [0.80-1.85]	3.52E-01	I	0.623	0.434	0.450	2.18 [1.63-2.93]	1.93E-07	I	0.598	0.447	0.464	1.86 [1.49-2.31]	3.72E-08
rs7720488	5	50746191	A/G	0.421	0.266	0.288	2.39 [1.37-4.17]	2.26E-03	I	0.300	0.238	0.248	1.40 [0.88-2.22]	1.59E-01	I	0.399	0.251	0.264	1.97 [1.48-2.64]	4.24E-06	I	0.377	0.251	0.265	1.88 [1.50-2.35]	4.15E-08
rs9291777	5	50742575	T/C	0.422	0.266	0.288	2.39 [1.37-4.18]	2.18E-03	I	0.300	0.238	0.248	1.39 [0.88-2.22]	1.62E-01	-	0.399	0.251	0.264	1.97 [1.48-2.63]	4.30E-06	I	0.377	0.252	0.265	1.88 [1.50-2.35]	4.19E-08
rs7722654	5	50740885	T/C	0.421	0.261	0.284	2.45 [1.40-4.28]	1.68E-03	I	0.296	0.237	0.247	1.37 [0.86-2.19]	1.85E-01	I	0.395	0.248	0.261	1.98 [1.48-2.64]	4.27E-06	I	0.374	0.248	0.262	1.88 [1.50-2.36]	4.23E-08
rs60195170	5	50745323	T/C	0.419	0.262	0.285	2.43 [1.39-4.25]	1.85E-03	I	0.296	0.237	0.247	1.38 [0.86-2.20]	1.79E-01	I	0.395	0.248	0.260	1.98 [1.48-2.64]	4.33E-06	I	0.374	0.248	0.262	1.88 [1.50-2.35]	4.38E-08
rs4865668	5	50748359	G/A	0.420	0.266	0.288	2.38 [1.36-4.16]	2.37E-03	I	0.300	0.238	0.249	1.39 [0.87-2.22]	1.62E-01	I	0.399	0.251	0.264	1.97 [1.48-2.64]	4.25E-06	I	0.377	0.251	0.265	1.88 [1.50-2.35]	4.41E-08
rs9885436	5	50764649	A/G	0.400	0.265	0.284	2.24 [1.28-3.95]	5.01E-03	I	0.308	0.238	0.250	1.45 [0.91-2.31]	1.15E-01	I	0.399	0.252	0.264	1.96 [1.47-2.62]	4.76E-06	I	0.375	0.252	0.265	1.87 [1.49-2.34]	5.17E-08
rs10471549	5	50665962	G/A	0.430	0.266	0.289	2.48 [1.42-4.31]	1.35E-03	I	0.299	0.243	0.253	1.34 [0.85-2.11]	2.14E-01	I	0.393	0.247	0.259	1.96 [1.47-2.61]	4.67E-06	I	0.376	0.249	0.263	1.86 [1.49-2.32]	5.25E-08
rs3811911	5	50684451	T/C	0.435	0.270	0.293	2.51 [1.44-4.38]	1.20E-03	I	0.300	0.243	0.253	1.34 [0.85-2.11]	2.12E-01	-	0.395	0.248	0.261	1.95 [1.46-2.60]	5.05E-06	-	0.378	0.251	0.265	1.85 [1.48-2.32]	5.26E-08
rs7448043	5	50768011	C/T	0.398	0.264	0.283	2.23 [1.27-3.93]	5.33E-03	I	0.308	0.238	0.250	1.46 [0.92-2.32]	1.12E-01	I	0.398	0.251	0.264	1.96 [1.47-2.62]	4.80E-06	I	0.375	0.251	0.265	1.87 [1.49-2.34]	5.28E-08
rs2161592	5	50772554	T/C	0.399	0.264	0.283	2.25 [1.28-3.97]	5.00E-03	I	0.309	0.238	0.250	1.46 [0.92-2.32]	1.10E-01	I	0.398	0.251	0.264	1.96 [1.47-2.61]	5.17E-06	I	0.375	0.251	0.265	1.87 [1.49-2.34]	5.30E-08
rs2161591	5	50772920	A/G	0.399	0.264	0.283	2.25 [1.28-3.97]	4.94E-03	I	0.309	0.238	0.250	1.46 [0.92-2.32]	1.10E-01	I	0.398	0.251	0.264	1.95 [1.46-2.61]	5.42E-06	I	0.374	0.251	0.265	1.87 [1.49-2.34]	5.51E-08
rs11747876	5	50773488	T/A	0.399	0.264	0.283	2.25 [1.28-3.97]	4.93E-03	I	0.309	0.238	0.250	1.46 [0.92-2.32]	1.10E-01	I	0.398	0.252	0.264	1.95 [1.46-2.61]	5.45E-06	I	0.374	0.251	0.265	1.87 [1.49-2.34]	5.51E-08
rs9291769	5	50717827	G/A	0.562	0.419	0.440	1.88 [1.10-3.21]	2.01E-02	I	0.454	0.399	0.409	1.26 [0.81-1.95]	3.07E-01	I	0.560	0.383	0.398	2.25 [1.66-3.04]	1.58E-07	I	0.533	0.391	0.406	1.87 [1.49-2.34]	6.08E-08
rs10075800	5	50659190	A/C	0.431	0.269	0.292	2.46 [1.41-4.29]	1.50E-03	I	0.300	0.243	0.253	1.34 [0.85-2.11]	2.13E-01	I	0.395	0.249	0.261	1.95 [1.46-2.60]	5.33E-06	I	0.377	0.251	0.265	1.85 [1.48-2.31]	6.29E-08
rs10056724	5	50665516	G/A	0.430	0.269	0.292	2.44 [1.40-4.25]	1.60E-03	I	0.299	0.243	0.253	1.33 [0.85-2.10]	2.16E-01	I	0.395	0.249	0.261	1.95 [1.46-2.60]	5.27E-06	I	0.377	0.251	0.265	1.85 [1.48-2.31]	6.66E-08
rs10055984	5	50663550	T/C	0.430	0.269	0.292	2.44 [1.40-4.26]	1.60E-03	I	0.299	0.243	0.253	1.33 [0.85-2.10]	2.16E-01	I	0.395	0.249	0.261	1.95 [1.46-2.60]	5.44E-06	I	0.377	0.251	0.265	1.85 [1.48-2.31]	6.85E-08
rs2059224	5	50777596	A/G	0.399	0.265	0.284	2.26 [1.28-3.99]	4.91E-03	I	0.309	0.238	0.250	1.46 [0.92-2.33]	1.08E-01	I	0.398	0.254	0.267	1.94 [1.45-2.59]	7.06E-06	I	0.375	0.253	0.267	1.86 [1.48-2.33]	6.86E-08
rs28454398	5	50793259	G/T	0.629	0.511	0.527	1.60 [0.96-2.68]	7.27E-02	I	0.550	0.474	0.487	1.37 [0.90-2.07]	1.42E-01	I	0.652	0.458	0.474	2.19 [1.63-2.95]	1.74E-07	I	0.62	0.468	0.485	1.82 [1.46-2.26]	6.96E-08

				GWAS1- subgroup A						GWAS1 - subgroup B						GWAS2						Meta-analysis				
SNP	Chr	Position	Risk/ other allel	RAF cases	RAF controls	RAF combined	RR [95% CI]	*P* value	Imputed	RAF cases	RAF controls	RAF combined	RR [95% CI]	*P* value	Imputed	RAF cases	RAF controls	RAF combined	RR [95% CI]	*P* value	Imputed	RAF cases	RAF controls	RAF combined	RR [95% CI]	*P* value
rs7737645	5	50796827	C/G	0.629	0.511	0.527	1.60 [0.96-2.68]	7.27E-02	I	0.550	0.474	0.487	1.37 [0.90-2.07]	1.41E-01	I	0.652	0.458	0.475	2.19 [1.63-2.94]	1.76E-07	I	0.62	0.469	0.485	1.82 [1.46-2.26]	7.00E-08
rs11960393	5	50781964	G/A	0.401	0.263	0.283	2.30 [1.30-4.07]	4.03E-03	I	0.309	0.238	0.250	1.46 [0.92-2.32]	1.09E-01	I	0.395	0.252	0.264	1.93 [1.44-2.57]	8.81E-06	I	0.373	0.251	0.265	1.86 [1.48-2.32]	7.41E-08
rs10053849	5	50781062	A/G	0.400	0.263	0.282	2.29 [1.30-4.04]	4.24E-03	I	0.309	0.238	0.250	1.46 [0.92-2.32]	1.10E-01	-	0.395	0.252	0.264	1.93 [1.44-2.57]	8.65E-06	-	0.374	0.251	0.265	1.85 [1.48-2.32]	7.68E-08
rs6899166	5	50781314	C/T	0.401	0.264	0.283	2.29 [1.30-4.04]	4.24E-03	I	0.309	0.238	0.250	1.46 [0.92-2.33]	1.07E-01	I	0.395	0.252	0.264	1.92 [1.44-2.57]	9.06E-06	I	0.373	0.251	0.265	1.85 [1.48-2.32]	7.75E-08
rs7708866	5	50676126	C/T	0.430	0.270	0.292	2.44 [1.40-4.25]	1.69E-03	I	0.300	0.245	0.254	1.33 [0.84-2.10]	2.20E-01	I	0.395	0.249	0.262	1.94 [1.46-2.59]	5.90E-06	I	0.377	0.252	0.265	1.84 [1.47-2.30]	7.91E-08
rs1559280	5	50780387	A/C	0.400	0.263	0.283	2.28 [1.29-4.03]	4.36E-03	I	0.309	0.238	0.250	1.46 [0.92-2.32]	1.09E-01	I	0.395	0.252	0.264	1.92 [1.44-2.57]	9.03E-06	I	0.373	0.251	0.265	1.85 [1.48-2.32]	8.02E-08
rs1559279	5	50781110	G/T	0.400	0.263	0.283	2.29 [1.30-4.04]	4.27E-03	I	0.309	0.238	0.250	1.46 [0.92-2.32]	1.09E-01	I	0.394	0.252	0.264	1.92 [1.44-2.56]	9.52E-06	I	0.373	0.251	0.264	1.85 [1.48-2.32]	8.31E-08
rs11954894	5	50674131	T/C	0.428	0.269	0.292	2.43 [1.39-4.23]	1.77E-03	I	0.309	0.257	0.266	1.31 [0.84-2.05]	2.34E-01	-	0.395	0.248	0.261	1.95 [1.46-2.60]	5.02E-06	I	0.379	0.253	0.267	1.83 [1.47-2.29]	8.38E-08
rs3792733	5	50682065	A/G	0.430	0.269	0.292	2.43 [1.40-4.24]	1.66E-03	I	0.300	0.247	0.256	1.31 [0.83-2.08]	2.42E-01	-	0.396	0.250	0.262	1.95 [1.46-2.59]	5.50E-06	I	0.378	0.252	0.266	1.84 [1.47-2.30]	8.45E-08
rs10053846	5	50612579	C/T	0.430	0.239	0.267	2.75 [1.56-4.88]	5.12E-04	-	0.264	0.219	0.227	1.32 [0.81-2.16]	2.72E-01	-	0.355	0.223	0.234	1.94 [1.44-2.62]	1.38E-05	-	0.346	0.225	0.238	1.89 [1.49-2.38]	9.82E-08
rs10060277	5	50791327	C/T	0.407	0.275	0.294	2.12 [1.21-3.73]	8.82E-03	I	0.324	0.239	0.253	1.57 [0.99-2.50]	5.46E-02	I	0.403	0.264	0.276	1.89 [1.41-2.52]	1.68E-05	I	0.383	0.262	0.275	1.84 [1.47-2.31]	1.01E-07
rs4538574	5	50743828	T/A	0.429	0.274	0.296	*2.60 [1.45-4.65]*	*1.27E-03*	I	0.303	0.242	0.253	1.38 [0.86-2.22]	1.76E-01	I	0.399	0.261	0.273	1.91 [1.43-2.56]	1.38E-05	I	0.38	0.26	0.273	1.86 [1.48-2.33]	1.04E-07
rs10037647	5	50612122	T/G	0.428	0.239	0.266	2.79 [1.57-4.97]	4.94E-04	I	0.261	0.216	0.224	1.33 [0.81-2.18]	2.64E-01	I	0.349	0.220	0.231	1.95 [1.44-2.64]	1.67E-05	I	0.342	0.222	0.235	1.90 [1.50-2.40]	1.10E-07
rs6867226	5	50705034	T/C	0.430	0.271	0.294	2.29 [1.33-3.94]	2.92E-03	I	0.300	0.247	0.256	1.32 [0.83-2.09]	2.36E-01	I	0.397	0.252	0.264	1.95 [1.46-2.60]	5.79E-06	I	0.378	0.254	0.267	1.83 [1.46-2.28]	1.18E-07
rs876510	5	50617759	T/C	0.430	0.242	0.268	2.75 [1.55-4.87]	5.37E-04	I	0.264	0.222	0.229	1.30 [0.79-2.13]	2.97E-01	I	0.355	0.224	0.235	1.94 [1.44-2.62]	1.43E-05	I	0.346	0.226	0.239	1.88 [1.49-2.38]	1.21E-07
rs6449538	5	50582954	T/C	0.429	0.240	0.267	2.77 [1.56-4.91]	5.12E-04	I	0.256	0.220	0.226	1.26 [0.77-2.07]	3.64E-01	I	0.352	0.223	0.234	1.96 [1.45-2.66]	1.27E-05	I	0.343	0.225	0.238	1.88 [1.49-2.38]	1.45E-07
rs66507130	5	50792240	A/G	0.407	0.276	0.295	2.12 [1.20-3.72]	9.15E-03	I	0.327	0.245	0.259	1.54 [0.97-2.43]	6.62E-02	I	0.404	0.267	0.278	1.86 [1.40-2.49]	2.20E-05	I	0.384	0.265	0.278	1.82 [1.45-2.27]	1.69E-07
rs72763871	5	50792294	A/T	0.405	0.273	0.291	2.15 [1.22-3.78]	7.93E-03	I	0.323	0.244	0.257	1.52 [0.96-2.41]	7.62E-02	I	0.399	0.263	0.274	1.87 [1.40-2.50]	2.28E-05	I	0.38	0.261	0.274	1.82 [1.45-2.28]	1.87E-07
rs1423611	5	50771114	T/G	0.698	0.573	0.591	1.82 [1.06-3.15]	3.10E-02	-	0.572	0.560	0.562	1.03 [0.68-1.56]	8.91E-01	I	0.759	0.541	0.560	2.67 [1.93-3.71]	3.64E-09	-	0.697	0.549	0.565	1.85 [1.47-2.34]	2.06E-07
rs12725009	1	3413997	A/G							0.027	0.019	0.020	1.80 [0.46-7.10]	3.99E-01	-	0.082	0.016	0.022	5.43 [2.91-10.15]	*1.13E-07*	-	0.054	0.015	0.019	*4.46 [2.54-7.94]*	2.27E-07
rs7734399	5	50791118	A/G	0.387	0.264	0.282	*2.10 [1.19-3.71]*	*1.06E-02*	I	0.309	0.237	0.249	*1.50 [0.93-2.41]*	*9.74E-02*	I	0.393	0.255	0.267	1.89 [1.41-2.53]	1.83E-05	I	0.369	0.254	0.266	*1.82 [1.45-2.29]*	*2.41E-07*
rs7446508	5	50791584	G/A	0.407	0.276	0.295	2.12 [1.21-3.72]	9.03E-03	-	0.327	0.245	0.259	1.54 [0.97-2.43]	6.66E-02	-	0.405	0.270	0.281	1.84 [1.38-2.46]	3.31E-05	-	0.385	0.267	0.28	1.80 [1.44-2.26]	2.49E-07
rs1364000	5	50767349	A/G	0.699	0.573	0.591	1.83 [1.06-3.17]	2.96E-02	I	0.572	0.561	0.563	1.03 [0.68-1.56]	9.04E-01	I	0.760	0.544	0.562	2.65 [1.91-3.68]	5.16E-09	I	0.697	0.551	0.567	1.84 [1.46-2.33]	2.60E-07
rs2161593	5	50766915	A/T	0.699	0.573	0.591	1.84 [1.06-3.17]	2.94E-02	I	0.572	0.561	0.563	1.02 [0.68-1.55]	9.12E-01	I	0.760	0.544	0.563	2.65 [1.91-3.68]	5.24E-09	I	0.697	0.551	0.567	1.84 [1.46-2.33]	2.70E-07
rs11580861	1	194995131	T/C	0.073	0.053	0.056	*2.40 [0.82-7.03]*	*1.09E-01*	I	0.044	0.020	0.024	2.74 [0.80-9.42]	1.09E-01	I	0.093	0.029	0.034	3.73 [2.14-6.52]	3.66E-06	I	0.076	0.031	0.036	*3.30 [2.08-5.22]*	*3.49E-07*
rs6891554	5	50784546	A/C	0.698	0.573	0.591	1.84 [1.06-3.17]	2.91E-02	I	0.573	0.560	0.562	1.03 [0.68-1.56]	8.72E-01	-	0.755	0.545	0.563	2.58 [1.86-3.57]	1.06E-08	-	0.695	0.551	0.567	1.82 [1.45-2.30]	3.55E-07
rs2059223	5	50778482	A/T	0.698	0.573	0.591	1.83 [1.06-3.16]	2.95E-02	I	0.573	0.561	0.563	1.03 [0.68-1.56]	8.87E-01	I	0.757	0.546	0.564	2.59 [1.87-3.59]	9.89E-09	I	0.696	0.552	0.568	1.83 [1.45-2.30]	3.67E-07
rs17824958	5	50779044	A/G	0.698	0.573	0.591	1.83 [1.06-3.16]	2.94E-02	I	0.573	0.560	0.563	1.03 [0.68-1.56]	8.85E-01	I	0.757	0.546	0.564	2.59 [1.87-3.58]	1.04E-08	I	0.696	0.552	0.568	1.82 [1.45-2.30]	3.76E-07
rs62505227	8	40287249	A/G	0.201	0.080	0.098	*3.40 [1.60-7.21]*	*1.45E-03*	I	0.127	0.067	0.077	2.18 [1.08-4.40]	2.95E-02	I	0.165	0.091	0.098	2 [1.35-2.98]	5.87E-04	I	0.162	0.086	0.094	*2.23 [1.63-3.06]*	*5.14E-07*
rs2062721	5	50599816	T/G	0.430	0.256	0.281	2.51 [1.44-4.38]	1.22E-03	I	0.263	0.241	0.245	1.17 [0.72-1.91]	5.30E-01	I	0.361	0.229	0.240	1.95 [1.44-2.64]	1.33E-05	I	0.35	0.235	0.247	1.81 [1.44-2.29]	5.19E-07
rs6449586	5	50634878	T/C	0.710	0.593	0.610	*1.73 [0.97-3.09]*	*6.53E-02*	I	0.609	0.577	0.582	1.18 [0.77-1.80]	4.45E-01	-	0.745	0.559	0.575	2.37 [1.72-3.28]	1.58E-07	-	0.702	0.567	0.582	*1.82 [1.44-2.30]*	*6.37E-07*
rs12332270	5	50776171	T/C	0.687	0.570	0.587	1.76 [1.02-3.03]	4.06E-02	I	0.565	0.560	0.560	1.00 [0.66-1.52]	9.89E-01	I	0.746	0.540	0.557	2.52 [1.83-3.48]	1.80E-08	I	0.686	0.548	0.563	1.78 [1.41-2.24]	9.26E-07
rs78034465	1	194732879	A/G	0.070	0.053	0.055	1.88 [0.69-5.15]	2.18E-01	I	0.057	0.031	0.036	1.95 [0.79-4.81]	1.46E-01	I	0.103	0.033	0.039	3.66 [2.14-6.26]	2.17E-06	I	0.084	0.036	0.041	2.85 [1.87-4.33]	1.01E-06
rs75182938	1	194732834	C/T	0.070	0.053	0.055	1.88 [0.69-5.15]	2.18E-01	I	0.057	0.031	0.036	1.95 [0.79-4.81]	1.46E-01	I	0.103	0.033	0.039	3.66 [2.14-6.25]	2.18E-06	I	0.084	0.036	0.041	2.85 [1.87-4.33]	1.01E-06
rs148540302	1	194727060	A/G	0.070	0.053	0.055	1.89 [0.69-5.16]	2.16E-01	I	0.057	0.031	0.036	1.95 [0.79-4.80]	1.46E-01	I	0.104	0.033	0.039	3.65 [2.14-6.24]	2.19E-06	I	0.084	0.036	0.041	2.84 [1.87-4.32]	1.01E-06
rs10052591	5	50812738	C/T	0.640	0.494	0.514	1.87 [1.10-3.18]	2.07E-02	I	0.473	0.431	0.438	1.20 [0.80-1.80]	3.90E-01	I	0.605	0.436	0.451	1.97 [1.48-2.62]	3.03E-06	I	0.577	0.444	0.459	1.70 [1.38-2.11]	1.02E-06
rs10119066	9	7509895	T/G	0.097	0.052	0.058	1.69 [0.68-4.25]	2.61E-01	I	0.063	0.053	0.054	1.45 [0.58-3.65]	4.30E-01	I	0.148	0.059	0.067	3.15 [2.01-4.93]	5.75E-07	I	0.115	0.057	0.063	2.51 [1.74-3.64]	1.04E-06
rs6449584	5	50632719	C/T	0.712	0.600	0.616	*1.71 [0.95-3.08]*	*7.17E-02*	I	0.633	0.590	0.598	1.27 [0.81-1.99]	2.92E-01	I	0.741	0.573	0.588	2.21 [1.61-3.05]	1.28E-06	I	0.707	0.58	0.594	*1.81 [1.43-2.30]*	*1.04E-06*
rs1475601	1	194721655	G/A	0.070	0.053	0.055	1.89 [0.69-5.16]	2.15E-01	I	0.057	0.031	0.036	1.95 [0.79-4.80]	1.46E-01	I	0.103	0.033	0.039	3.64 [2.13-6.23]	2.27E-06	I	0.084	0.036	0.041	2.84 [1.87-4.32]	1.04E-06
rs187824709	1	194705694	T/C	0.070	0.052	0.055	1.9 [0.70-5.20]	2.10E-01	I	0.057	0.031	0.036	1.94 [0.79-4.79]	1.48E-01	I	0.104	0.033	0.039	3.62 [2.12-6.17]	2.41E-06	I	0.085	0.036	0.041	2.83 [1.86-4.30]	1.05E-06
rs2068701	5	50814632	G/A	0.640	0.494	0.515	1.87 [1.10-3.18]	2.09E-02	-	0.473	0.431	0.438	1.2 [0.80-1.80]	3.88E-01	-	0.604	0.436	0.451	1.97 [1.48-2.61]	3.15E-06	I	0.577	0.444	0.459	1.7 [1.38-2.11]	1.05E-06
rs6864170	5	50757865	A/T	0.414	0.286	0.304	1.94 [1.14-3.30]	1.50E-02	I	0.300	0.245	0.254	1.37 [0.85-2.18]	1.93E-01	I	0.396	0.262	0.273	1.87 [1.40-2.50]	2.70E-05	I	0.374	0.263	0.275	1.75 [1.40-2.19]	1.06E-06
rs10061466	5	50741972	T/C	0.422	0.289	0.308	1.96 [1.15-3.32]	1.30E-02	I	0.300	0.249	0.258	1.32 [0.83-2.09]	2.38E-01	I	0.400	0.265	0.276	1.87 [1.40-2.50]	2.35E-05	I	0.378	0.266	0.278	1.74 [1.39-2.17]	1.16E-06
rs410285	22	22381768	G/A													0.055	0.010	0.014	6.45 [3.04-13.67]	1.17E-06	-	0.031	0.011	0.014	6.45 [3.04-13.67]	1.17E-06
rs2217315	5	50656817	A/C	0.541	0.354	0.381	2.49 [1.41-4.41]	*1.77E-03*	I	0.366	0.354	0.356	1.09 [0.68-1.76]	*7.17E-01*	I	0.476	0.332	0.344	1.95 [1.45-2.63]	*1.09E-05*	I	0.461	0.339	0.352	1.77 [1.41-2.23]	*1.22E-06*
rs78315000	8	69103058	G/A	0.070	0.041	0.045	3.21 [0.77-13.28]	*1.08E-01*	I	0.061	0.042	0.045	2.13 [0.66-6.88]	*2.05E-01*	I	0.090	0.042	0.047	4.86 [2.46-9.60]	*5.23E-06*	I	0.078	0.042	0.046	3.83 [2.22-6.60]	*1.28E-06*
rs78000786	1	194707918	T/C	0.070	0.054	0.056	1.88 [0.69-5.13]	2.20E-01	I	0.057	0.032	0.036	1.95 [0.79-4.79]	1.48E-01	I	0.104	0.034	0.040	3.59 [2.10-6.14]	2.89E-06	I	0.085	0.036	0.042	2.81 [1.85-4.27]	1.30E-06
rs80171669	1	194714561	A/G	0.070	0.052	0.055	1.9 [0.70-5.19]	2.10E-01	I	0.057	0.031	0.036	1.95 [0.79-4.79]	1.47E-01	I	0.103	0.033	0.039	3.58 [2.09-6.12]	3.10E-06	I	0.084	0.036	0.041	2.81 [1.85-4.27]	1.30E-06
rs7719442	5	50743281	G/A	0.422	0.289	0.308	1.96 [1.15-3.32]	1.31E-02	I	0.300	0.249	0.258	1.32 [0.83-2.10]	2.38E-01	-	0.400	0.266	0.277	1.86 [1.39-2.48]	2.82E-05	-	0.378	0.267	0.279	1.73 [1.39-2.16]	1.36E-06
rs79876351	1	194707687	C/G	0.070	0.052	0.055	1.9 [0.70-5.20]	2.10E-01	I	0.057	0.031	0.036	1.94 [0.79-4.79]	1.48E-01	I	0.103	0.033	0.039	3.56 [2.08-6.08]	3.45E-06	I	0.084	0.036	0.041	2.8 [1.84-4.26]	1.42E-06
rs9283702	5	50755613	G/C	0.418	0.290	0.308	1.92 [1.13-3.26]	1.63E-02	I	0.302	0.249	0.258	1.33 [0.84-2.12]	2.26E-01	I	0.399	0.266	0.277	1.86 [1.39-2.49]	3.03E-05	I	0.377	0.267	0.279	1.73 [1.38-2.16]	1.60E-06
rs17339448	5	50596500	G/A	0.211	0.101	0.117	2.4 [1.24-4.62]	9.05E-03	I	0.146	0.097	0.105	1.69 [0.88-3.24]	1.15E-01	I	0.186	0.102	0.109	2.07 [1.42-3.01]	1.60E-04	I	0.18	0.101	0.11	2.04 [1.53-2.74]	1.62E-06
rs113391436	1	194694847	T/G	0.070	0.052	0.055	1.9 [0.70-5.20]	2.09E-01	I	0.056	0.031	0.036	1.92 [0.78-4.74]	1.57E-01	I	0.103	0.033	0.039	3.54 [2.07-6.05]	3.86E-06	I	0.084	0.036	0.041	2.78 [1.83-4.23]	1.67E-06
rs10045593	5	50790356	T/C	0.418	0.274	0.294	2.38 [1.35-4.20]	2.74E-03	I	0.309	0.257	0.266	1.3 [0.82-2.05]	2.69E-01	I	0.394	0.270	0.280	1.78 [1.33-2.37]	9.25E-05	I	0.376	0.268	0.28	1.73 [1.38-2.16]	1.89E-06
rs192378410	6	168972558	T/C	0.025	0.025	0.025	1.85 [0.26-13.30]	*5.40E-01*	I	0.049	0.023	0.028	3.36 [0.90-12.61]	*7.26E-02*	I	0.076	0.026	0.030	4.78 [2.41-9.49]	*7.93E-06*	I	0.058	0.025	0.029	4.11 [2.30-7.35]	*1.94E-06*
rs56767241	5	50788266	A/C	0.418	0.275	0.295	2.37 [1.35-4.19]	2.82E-03	I	0.309	0.258	0.267	1.29 [0.82-2.04]	2.75E-01	I	0.395	0.271	0.281	1.78 [1.33-2.37]	9.20E-05	I	0.377	0.269	0.281	1.72 [1.38-2.16]	1.98E-06
rs9354654	6	68713399	C/T	0.441	0.380	0.611	1.32 [0.81-2.17]	2.68E-01	I	0.558	0.370	0.598	1.98 [1.30-3.04]	1.62E-03	I	0.504	0.375	0.614	1.68 [1.26-2.23]	3.36E-04	I	0.505	0.375	0.389	1.67 [1.35-2.07]	2.08E-06
rs76623541	1	194690362	A/G	0.070	0.052	0.055	1.9 [0.70-5.20]	2.09E-01	I	0.055	0.032	0.036	1.88 [0.76-4.65]	1.72E-01	I	0.102	0.033	0.039	3.5 [2.05-5.99]	4.75E-06	I	0.083	0.036	0.041	2.76 [1.81-4.19]	2.20E-06
rs115808189	5	50417563	G/A	0.055	0.034	0.037	2.04 [0.52-7.93]	*3.05E-01*	I	0.054	0.029	0.033	2.54 [0.79-8.20]	*1.19E-01*	I	0.091	0.037	0.042	3.99 [2.17-7.34]	*8.24E-06*	I	0.074	0.035	0.039	3.35 [2.03-5.54]	*2.32E-06*
rs80100429	1	194688249	G/A	0.070	0.052	0.055	1.9 [0.70-5.20]	2.09E-01	I	0.055	0.032	0.036	1.87 [0.76-4.63]	1.76E-01	I	0.102	0.034	0.039	3.49 [2.04-5.97]	5.03E-06	I	0.083	0.036	0.041	2.75 [1.81-4.18]	2.37E-06
rs12235761	9	7506363	A/G	0.191	0.141	0.148	1.63 [0.82-3.21]	1.61E-01	I	0.245	0.187	0.197	1.52 [0.92-2.52]	1.05E-01	I	0.296	0.168	0.179	2.03 [1.47-2.81]	1.53E-05	I	0.261	0.167	0.177	1.83 [1.43-2.36]	2.39E-06
rs2381621	9	7506117	T/G	0.191	0.141	0.148	1.62 [0.82-3.20]	1.62E-01	I	0.245	0.187	0.197	1.52 [0.92-2.52]	1.05E-01	I	0.296	0.168	0.179	2.03 [1.47-2.80]	1.59E-05	I	0.261	0.167	0.177	1.83 [1.42-2.36]	2.49E-06
rs78888493	1	194687185	C/A	0.070	0.052	0.055	1.9 [0.70-5.20]	2.09E-01	I	0.055	0.032	0.036	1.87 [0.75-4.62]	1.78E-01	I	0.102	0.034	0.039	3.48 [2.03-5.95]	5.29E-06	I	0.083	0.036	0.041	2.74 [1.80-4.17]	2.50E-06
rs76258079	1	194686792	T/C	0.070	0.052	0.055	1.9 [0.70-5.20]	2.09E-01	I	0.055	0.032	0.036	1.86 [0.75-4.62]	1.79E-01	I	0.102	0.034	0.039	3.48 [2.03-5.95]	5.34E-06	I	0.083	0.036	0.041	2.74 [1.80-4.17]	2.53E-06
rs5767218	22	46879314	C/A	0.415	0.376	0.619	1.32 [0.78-2.23]	3.08E-01	I	0.539	0.384	0.589	2.1 [1.33-3.29]	1.33E-03	I	0.478	0.362	0.628	1.7 [1.27-2.28]	*4.10E-04*	I	0.481	0.367	0.38	1.71 [1.37-2.14]	*2.60E-06*
rs1514351	6	68694074	C/T	0.442	0.386	0.394	1.30 [0.80-2.13]	2.89E-01	I	0.564	0.377	0.409	1.94 [1.28-2.96]	1.92E-03	I	0.508	0.379	0.390	1.67 [1.26-2.22]	3.52E-04	I	0.509	0.38	0.394	1.66 [1.34-2.05]	2.66E-06
rs10491412	5	50671121	T/C	0.198	0.098	0.113	2.36 [1.21-4.60]	1.17E-02	-	0.145	0.099	0.107	1.58 [0.84-2.98]	1.56E-01	-	0.191	0.105	0.112	2.05 [1.42-2.97]	1.45E-04	-	0.18	0.103	0.111	1.99 [1.49-2.66]	2.68E-06
rs113300181	1	194586278	A/G	0.070	0.052	0.055	1.90 [0.70-5.19]	2.10E-01	I	0.055	0.032	0.036	1.83 [0.74-4.55]	1.91E-01	I	0.100	0.033	0.039	3.55 [2.06-6.11]	5.04E-06	I	0.082	0.036	0.041	2.75 [1.80-4.20]	2.76E-06
rs115979981	1	194589377	T/C	0.070	0.052	0.055	1.90 [0.70-5.19]	2.10E-01	I	0.055	0.032	0.036	1.83 [0.74-4.55]	1.91E-01	I	0.101	0.033	0.039	3.55 [2.06-6.11]	5.05E-06	I	0.082	0.036	0.041	2.75 [1.80-4.20]	2.77E-06
rs75024310	1	194590891	T/G	0.070	0.052	0.055	1.90 [0.70-5.19]	2.10E-01	I	0.055	0.032	0.036	1.83 [0.74-4.55]	1.91E-01	I	0.101	0.033	0.039	3.54 [2.06-6.10]	5.06E-06	I	0.082	0.036	0.041	2.75 [1.80-4.20]	2.77E-06
rs7454982	6	68707736	A/G	0.442	0.386	0.606	1.31 [0.80-2.13]	2.88E-01	I	0.564	0.378	0.590	1.93 [1.27-2.95]	2.09E-03	I	0.509	0.379	0.610	1.67 [1.26-2.22]	3.45E-04	I	0.509	0.38	0.394	1.66 [1.34-2.05]	2.77E-06
rs7764047	6	68698932	C/T	0.442	0.386	0.394	1.30 [0.80-2.13]	2.89E-01	I	0.564	0.378	0.410	1.93 [1.27-2.95]	2.10E-03	I	0.509	0.379	0.390	1.67 [1.26-2.22]	3.45E-04	I	0.51	0.38	0.394	1.66 [1.34-2.05]	2.79E-06
rs12192207	6	68705650	T/C	0.442	0.386	0.394	1.31 [0.80-2.13]	2.88E-01	I	0.564	0.378	0.410	1.93 [1.27-2.95]	2.10E-03	I	0.509	0.379	0.390	1.67 [1.26-2.22]	3.48E-04	I	0.509	0.38	0.394	1.66 [1.34-2.05]	2.79E-06
rs1567904	6	68697721	A/G	0.442	0.386	0.394	1.30 [0.80-2.13]	2.91E-01	-	0.564	0.378	0.410	1.93 [1.27-2.95]	2.10E-03	I	0.509	0.379	0.390	1.67 [1.26-2.22]	3.45E-04	I	0.51	0.38	0.394	1.66 [1.34-2.05]	2.80E-06
rs1514352	6	68694302	T/G	0.442	0.386	0.394	1.31 [0.80-2.13]	2.88E-01	I	0.564	0.378	0.410	1.94 [1.27-2.95]	2.07E-03	I	0.509	0.379	0.390	1.67 [1.26-2.22]	3.51E-04	I	0.509	0.38	0.394	1.66 [1.34-2.05]	2.80E-06
rs6936599	6	68693943	T/A	0.442	0.386	0.394	1.31 [0.80-2.13]	2.88E-01	I	0.564	0.378	0.410	1.94 [1.27-2.95]	2.07E-03	I	0.509	0.379	0.390	1.67 [1.26-2.22]	3.52E-04	I	0.509	0.38	0.394	1.66 [1.34-2.05]	2.80E-06

				GWAS1- subgroup A						GWAS1 - subgroup B						GWAS2						Meta-analysis				
SNP	Chr	Position	Risk/ other allel	RAF cases	RAF controls	RAF combined	RR [95% CI]	*P* value	Imputed	RAF cases	RAF controls	RAF combined	RR [95% CI]	*P* value	Imputed	RAF cases	RAF controls	RAF combined	RR [95% CI]	*P* value	Imputed	RAF cases	RAF controls	RAF combined	RR [95% CI]	*P* value
rs6936435	6	68693857	G/A	0.442	0.386	0.394	1.31 [0.80-2.13]	2.88E-01	I	0.564	0.378	0.410	1.94 [1.27-2.95]	2.07E-03	I	0.509	0.379	0.390	1.67 [1.26-2.22]	3.52E-04	I	0.509	0.38	0.394	1.66 [1.34-2.05]	2.80E-06
rs7760421	6	68699179	G/A	0.442	0.386	0.394	1.30 [0.80-2.13]	2.89E-01	I	0.564	0.378	0.410	1.93 [1.27-2.95]	2.10E-03	I	0.509	0.379	0.391	1.67 [1.26-2.22]	3.49E-04	I	0.51	0.38	0.394	1.66 [1.34-2.05]	2.81E-06
rs2361763	16	85993134	C/T	0.623	0.526	0.461	1.66 [0.97-2.82]	*6.34E-02*	I	0.618	0.487	0.491	1.77 [1.12-2.79]	1.48E-02	-	0.618	0.489	0.500	1.72 [1.28-2.33]	3.89E-04	-	0.619	0.494	0.508	*1.72 [1.37-2.16]*	*2.82E-06*
rs113599876	1	194579022	A/G	0.069	0.043	0.046	2.51 [0.86-7.39]	9.34E-02	I	0.055	0.030	0.035	1.98 [0.79-4.99]	1.46E-01	I	0.095	0.033	0.038	3.34 [1.91-5.83]	2.27E-05	I	0.079	0.034	0.039	2.84 [1.83-4.39]	2.84E-06
rs76989778	1	194681828	T/C	0.070	0.052	0.055	1.90 [0.70-5.20]	2.08E-01	I	0.055	0.032	0.036	1.85 [0.75-4.59]	1.84E-01	I	0.102	0.034	0.039	3.45 [2.02-5.90]	6.04E-06	I	0.083	0.036	0.041	2.72 [1.79-4.14]	2.88E-06
rs12193625	6	68697160	G/A	0.442	0.386	0.394	1.30 [0.80-2.13]	2.89E-01	I	0.564	0.378	0.410	1.94 [1.27-2.95]	2.07E-03	-	0.509	0.380	0.391	1.67 [1.26-2.22]	3.67E-04	-	0.51	0.381	0.395	1.66 [1.34-2.05]	2.93E-06
rs75586085	1	194679772	A/G	0.070	0.052	0.055	1.91 [0.70-5.20]	2.08E-01	I	0.055	0.032	0.036	1.85 [0.75-4.59]	1.84E-01	I	0.102	0.034	0.039	3.45 [2.01-5.89]	6.29E-06	I	0.083	0.036	0.041	2.72 [1.79-4.14]	2.98E-06
rs10050915	5	50791335	T/C	0.698	0.599	0.613	1.59 [0.92-2.75]	9.55E-02	I	0.591	0.579	0.581	1.05 [0.69-1.60]	8.28E-01	I	0.769	0.573	0.590	2.48 [1.78-3.45]	7.11E-08	I	0.707	0.578	0.592	1.75 [1.38-2.21]	3.04E-06
rs11581126	1	194677323	A/G	0.070	0.052	0.055	1.91 [0.70-5.20]	2.08E-01	I	0.055	0.032	0.036	1.85 [0.75-4.59]	1.84E-01	I	0.101	0.034	0.039	3.44 [2.01-5.88]	6.59E-06	I	0.083	0.036	0.041	2.72 [1.78-4.13]	3.10E-06
rs4707315	6	68713027	G/A	0.442	0.385	0.607	1.30 [0.80-2.13]	2.89E-01	I	0.564	0.378	0.590	1.94 [1.27-2.95]	2.06E-03	I	0.507	0.379	0.610	1.66 [1.26-2.21]	4.00E-04	I	0.509	0.38	0.394	1.65 [1.34-2.04]	3.16E-06
rs9363844	6	68717074	A/T	0.441	0.386	0.607	1.30 [0.80-2.13]	2.95E-01	I	0.563	0.379	0.589	1.92 [1.26-2.92]	2.25E-03	I	0.510	0.381	0.608	1.67 [1.26-2.22]	3.68E-04	I	0.51	0.382	0.396	1.65 [1.34-2.05]	3.17E-06
rs12193697	6	68697317	T/A	0.442	0.386	0.394	1.30 [0.80-2.13]	2.89E-01	I	0.564	0.378	0.410	1.94 [1.27-2.95]	2.07E-03	I	0.507	0.379	0.390	1.66 [1.26-2.21]	4.03E-04	I	0.509	0.38	0.394	1.65 [1.34-2.04]	3.19E-06
rs7773398	6	68698751	T/G	0.442	0.386	0.394	1.30 [0.80-2.13]	2.89E-01	I	0.564	0.378	0.410	1.94 [1.27-2.95]	2.07E-03	I	0.507	0.379	0.390	1.66 [1.25-2.21]	4.04E-04	I	0.509	0.38	0.394	1.65 [1.34-2.04]	3.20E-06
rs2137887	6	68700632	T/C	0.442	0.386	0.394	1.30 [0.80-2.13]	2.89E-01	I	0.564	0.378	0.410	1.94 [1.27-2.95]	2.07E-03	I	0.507	0.379	0.390	1.66 [1.25-2.21]	4.05E-04	I	0.509	0.38	0.394	1.65 [1.34-2.04]	3.22E-06
rs75121132	1	194669496	C/T	0.070	0.052	0.055	1.90 [0.70-5.20]	2.08E-01	I	0.055	0.032	0.036	1.85 [0.75-4.59]	1.83E-01	I	0.101	0.034	0.039	3.43 [2.00-5.87]	7.02E-06	I	0.082	0.036	0.041	2.71 [1.78-4.12]	3.26E-06
rs1986733	9	7505902	A/G	0.186	0.141	0.147	1.56 [0.79-3.08]	1.98E-01	-	0.245	0.187	0.197	1.52 [0.92-2.52]	1.05E-01	-	0.295	0.168	0.179	2.02 [1.47-2.79]	1.73E-05	-	0.26	0.167	0.177	1.82 [1.41-2.34]	3.26E-06
rs2113077	5	50799442	G/A	0.676	0.592	0.604	1.43 [0.84-2.42]	1.87E-01	I	0.615	0.577	0.583	1.19 [0.77-1.83]	4.37E-01	I	0.751	0.567	0.583	2.33 [1.68-3.22]	3.60E-07	I	0.699	0.572	0.586	1.74 [1.38-2.20]	3.28E-06
rs9346111	6	68711870	T/C	0.442	0.386	0.606	1.30 [0.80-2.13]	2.95E-01	I	0.564	0.380	0.588	1.94 [1.27-2.96]	2.06E-03	I	0.509	0.381	0.608	1.67 [1.26-2.21]	4.02E-04	I	0.51	0.382	0.396	1.65 [1.34-2.05]	3.30E-06
rs55756134	8	40285030	A/G	0.314	0.197	0.214	1.67 [0.94-2.94]	7.83E-02	I	0.264	0.166	0.182	2.01 [1.18-3.40]	9.74E-03	I	0.310	0.206	0.214	1.73 [1.27-2.37]	4.99E-04	I	0.298	0.198	0.209	1.78 [1.39-2.26]	3.31E-06
rs74613160	1	194667188	T/C	0.070	0.052	0.055	1.90 [0.70-5.19]	2.10E-01	I	0.055	0.032	0.036	1.85 [0.75-4.59]	1.84E-01	I	0.101	0.034	0.039	3.42 [2.00-5.86]	7.15E-06	I	0.082	0.036	0.041	2.71 [1.78-4.12]	3.35E-06
rs57086087	8	40301811	C/T	0.266	0.160	0.175	1.70 [0.93-3.09]	8.40E-02	I	0.209	0.128	0.142	1.96 [1.10-3.47]	2.18E-02	I	0.257	0.159	0.167	1.87 [1.34-2.61]	2.42E-04	I	0.246	0.154	0.164	1.85 [1.43-2.40]	3.36E-06
rs10993625	9	93442205	C/G	0.206	0.148	0.157	1.57 [0.81-3.04]	*1.79E-01*	I	0.200	0.168	0.173	*1.43 [0.77-2.64]*	*2.54E-01*	I	0.246	0.144	0.153	*2.38 [1.64-3.46]*	*4.64E-06*	I	0.226	0.149	0.157	*1.97 [1.48-2.63]*	*3.40E-06*
rs145025725	1	194665656	A/G	0.070	0.052	0.055	1.90 [0.70-5.18]	2.11E-01	I	0.055	0.032	0.036	1.85 [0.75-4.58]	1.84E-01	I	0.101	0.034	0.039	3.42 [2.00-5.86]	7.25E-06	I	0.082	0.036	0.041	2.71 [1.78-4.12]	3.41E-06
rs11589586	1	194599864	T/A	0.070	0.052	0.055	1.90 [0.70-5.19]	2.10E-01	I	0.055	0.032	0.036	1.85 [0.75-4.58]	1.85E-01	I	0.100	0.033	0.039	3.46 [2.01-5.96]	7.26E-06	I	0.082	0.036	0.041	2.72 [1.78-4.15]	3.52E-06
rs78250288	1	194599344	C/T	0.070	0.052	0.055	1.90 [0.70-5.19]	2.10E-01	I	0.055	0.032	0.036	1.84 [0.74-4.57]	1.87E-01	I	0.100	0.033	0.039	3.46 [2.01-5.96]	7.28E-06	I	0.082	0.036	0.041	2.72 [1.78-4.14]	3.59E-06
rs74644212	1	194600797	C/A	0.070	0.052	0.055	1.90 [0.70-5.18]	2.10E-01	I	0.055	0.032	0.036	1.84 [0.74-4.57]	1.87E-01	I	0.100	0.033	0.039	3.46 [2.01-5.95]	7.41E-06	I	0.082	0.036	0.041	2.71 [1.78-4.14]	3.64E-06
rs80146577	1	194602328	T/C	0.070	0.052	0.055	1.90 [0.70-5.18]	2.10E-01	I	0.055	0.032	0.036	1.84 [0.74-4.57]	1.87E-01	I	0.100	0.033	0.039	3.45 [2.01-5.94]	7.53E-06	I	0.082	0.036	0.041	2.71 [1.78-4.14]	3.68E-06
rs1354180	5	50587894	A/G	0.381	0.199	0.225	3.07 [1.69-5.58]	2.39E-04	I	0.232	0.185	0.193	1.38 [0.83-2.30]	2.17E-01	I	0.279	0.189	0.197	1.70 [1.24-2.35]	1.16E-03	I	0.288	0.19	0.201	1.79 [1.40-2.30]	3.76E-06
rs7451539	6	68709644	T/A	0.442	0.386	0.606	1.30 [0.80-2.12]	2.95E-01	I	0.564	0.380	0.589	1.92 [1.26-2.91]	2.35E-03	I	0.509	0.381	0.608	1.66 [1.25-2.20]	4.44E-04	I	0.51	0.382	0.396	1.65 [1.33-2.03]	3.93E-06
rs116153206	1	194579963	C/T	0.069	0.043	0.046	2.52 [0.86-7.40]	9.24E-02	I	0.055	0.030	0.034	1.98 [0.79-4.98]	1.47E-01	I	0.093	0.033	0.038	3.27 [1.87-5.72]	3.30E-05	I	0.078	0.034	0.039	2.80 [1.81-4.33]	3.94E-06
rs76822031	1	194575415	T/G	0.069	0.043	0.046	2.52 [0.86-7.41]	9.25E-02	I	0.055	0.030	0.034	1.98 [0.79-4.99]	1.46E-01	I	0.093	0.033	0.038	3.27 [1.87-5.72]	3.33E-05	I	0.078	0.034	0.039	2.80 [1.81-4.34]	3.94E-06
rs111328574	1	194659065	A/C	0.070	0.052	0.055	1.90 [0.70-5.18]	2.11E-01	I	0.055	0.032	0.036	1.84 [0.74-4.57]	1.87E-01	I	0.101	0.034	0.039	3.39 [1.98-5.81]	8.53E-06	I	0.082	0.036	0.041	2.69 [1.77-4.09]	3.98E-06
rs12205928	6	68708175	T/A	0.442	0.386	0.606	1.30 [0.79-2.12]	2.98E-01	I	0.564	0.380	0.589	1.92 [1.26-2.91]	2.35E-03	I	0.509	0.381	0.608	1.66 [1.25-2.20]	4.45E-04	I	0.51	0.382	0.396	1.64 [1.33-2.03]	4.00E-06
rs1514356	6	68700302	T/G	0.442	0.387	0.394	1.30 [0.80-2.12]	2.96E-01	I	0.564	0.380	0.411	1.91 [1.26-2.91]	2.35E-03	I	0.509	0.381	0.392	1.66 [1.25-2.20]	4.51E-04	I	0.51	0.382	0.396	1.64 [1.33-2.03]	4.01E-06
rs74906708	1	194605261	A/G	0.070	0.052	0.055	1.90 [0.70-5.18]	2.10E-01	I	0.055	0.032	0.036	1.84 [0.74-4.56]	1.86E-01	I	0.101	0.034	0.040	3.40 [1.98-5.82]	8.70E-06	I	0.082	0.036	0.041	2.69 [1.77-4.09]	4.05E-06
rs1514354	6	68705751	G/A	0.442	0.386	0.394	1.30 [0.79-2.12]	2.98E-01	I	0.564	0.380	0.411	1.91 [1.26-2.91]	2.35E-03	I	0.509	0.381	0.392	1.66 [1.25-2.20]	4.50E-04	I	0.51	0.382	0.396	1.64 [1.33-2.03]	4.05E-06
rs1355931	6	68705526	G/A	0.442	0.386	0.394	1.30 [0.79-2.12]	2.98E-01	I	0.564	0.380	0.411	1.91 [1.26-2.91]	2.35E-03	-	0.509	0.382	0.393	1.66 [1.25-2.20]	4.54E-04	-	0.51	0.382	0.396	1.64 [1.33-2.03]	4.07E-06
rs11582052	1	194608177	A/C	0.070	0.052	0.055	1.90 [0.70-5.18]	2.10E-01	I	0.055	0.032	0.036	1.84 [0.74-4.56]	1.87E-01	I	0.101	0.034	0.040	3.39 [1.98-5.82]	8.79E-06	I	0.082	0.036	0.041	2.69 [1.76-4.09]	4.08E-06
rs10945046	6	68695436	C/T	0.442	0.387	0.395	1.30 [0.79-2.12]	2.98E-01	I	0.564	0.380	0.411	1.92 [1.26-2.91]	2.34E-03	I	0.509	0.381	0.392	1.66 [1.25-2.20]	4.57E-04	I	0.51	0.382	0.396	1.64 [1.33-2.03]	4.09E-06
rs74556709	1	194603121	A/G	0.070	0.052	0.055	1.90 [0.70-5.18]	2.11E-01	I	0.055	0.032	0.036	1.84 [0.74-4.55]	1.89E-01	I	0.101	0.034	0.040	3.40 [1.98-5.82]	8.70E-06	I	0.082	0.036	0.041	2.69 [1.76-4.09]	4.12E-06
rs11577001	1	194603864	C/T	0.070	0.052	0.055	1.90 [0.70-5.18]	2.10E-01	I	0.055	0.032	0.036	1.83 [0.74-4.53]	1.89E-01	-	0.101	0.034	0.040	3.40 [1.98-5.82]	8.65E-06	I	0.082	0.036	0.041	2.69 [1.76-4.09]	4.13E-06
rs11578475	1	194611972	C/T	0.070	0.052	0.055	1.90 [0.70-5.18]	2.10E-01	I	0.055	0.032	0.036	1.84 [0.74-4.56]	1.86E-01	I	0.101	0.034	0.040	3.39 [1.98-5.81]	8.92E-06	I	0.082	0.036	0.041	2.69 [1.76-4.09]	4.13E-06
rs186202255	1	194727370	G/A	0.044	0.046	0.046	1.32 [0.38-4.51]	6.62E-01	I	0.040	0.022	0.025	2.19 [0.67-7.17]	1.95E-01	I	0.093	0.028	0.034	3.90 [2.21-6.88]	2.53E-06	I	0.069	0.03	0.034	3.03 [1.89-4.86]	4.14E-06
rs11577016	1	194603950	C/T	0.070	0.052	0.055	1.90 [0.70-5.18]	2.11E-01	I	0.055	0.032	0.036	1.84 [0.74-4.55]	1.89E-01	I	0.101	0.034	0.040	3.39 [1.98-5.82]	8.75E-06	I	0.082	0.036	0.041	2.69 [1.76-4.09]	4.14E-06
rs77231917	1	194615346	A/T	0.070	0.052	0.055	1.90 [0.70-5.18]	2.10E-01	I	0.055	0.032	0.036	1.84 [0.74-4.56]	1.86E-01	I	0.101	0.034	0.040	3.39 [1.98-5.81]	8.96E-06	I	0.082	0.036	0.041	2.68 [1.76-4.09]	4.14E-06
rs1913480	6	68695001	T/A	0.442	0.386	0.394	1.30 [0.80-2.12]	2.96E-01	I	0.564	0.380	0.412	1.92 [1.26-2.91]	2.34E-03	I	0.509	0.382	0.393	1.66 [1.25-2.20]	4.68E-04	I	0.51	0.382	0.396	1.64 [1.33-2.03]	4.14E-06
rs111838454	1	194619407	A/G	0.070	0.052	0.055	1.90 [0.70-5.18]	2.10E-01	I	0.055	0.032	0.036	1.84 [0.74-4.56]	1.86E-01	I	0.101	0.034	0.040	3.39 [1.98-5.80]	9.02E-06	I	0.082	0.036	0.041	2.68 [1.76-4.09]	4.16E-06
rs115777073	1	194620049	T/C	0.070	0.052	0.055	1.90 [0.70-5.18]	2.10E-01	I	0.055	0.032	0.036	1.84 [0.74-4.56]	1.86E-01	I	0.101	0.034	0.040	3.39 [1.98-5.80]	9.03E-06	I	0.082	0.036	0.041	2.68 [1.76-4.09]	4.17E-06
rs112273826	1	194652311	T/A	0.070	0.052	0.055	1.90 [0.70-5.18]	2.11E-01	I	0.055	0.032	0.036	1.84 [0.74-4.57]	1.86E-01	I	0.100	0.034	0.039	3.39 [1.98-5.80]	9.06E-06	I	0.082	0.036	0.041	2.68 [1.76-4.09]	4.18E-06
rs77617149	1	194607044	C/T	0.070	0.052	0.055	1.90 [0.70-5.18]	2.11E-01	I	0.055	0.032	0.036	1.84 [0.74-4.55]	1.89E-01	I	0.101	0.034	0.040	3.39 [1.98-5.81]	8.88E-06	I	0.082	0.036	0.041	2.68 [1.76-4.09]	4.19E-06
rs111261272	1	194622317	T/G	0.070	0.052	0.055	1.90 [0.70-5.18]	2.10E-01	I	0.055	0.032	0.036	1.84 [0.74-4.56]	1.87E-01	I	0.101	0.034	0.040	3.39 [1.98-5.80]	9.07E-06	I	0.082	0.036	0.041	2.68 [1.76-4.09]	4.19E-06
rs11582050	1	194608134	T/C	0.070	0.052	0.055	1.90 [0.70-5.18]	2.11E-01	I	0.055	0.032	0.036	1.84 [0.74-4.55]	1.89E-01	I	0.101	0.034	0.040	3.39 [1.98-5.81]	8.90E-06	I	0.082	0.036	0.041	2.68 [1.76-4.09]	4.20E-06
rs79731935	1	194608667	T/C	0.070	0.052	0.055	1.90 [0.70-5.18]	2.11E-01	I	0.055	0.032	0.036	1.84 [0.74-4.55]	1.89E-01	I	0.101	0.034	0.040	3.39 [1.98-5.81]	8.93E-06	I	0.082	0.036	0.041	2.68 [1.76-4.09]	4.21E-06
rs112398155	1	194609119	A/C	0.070	0.052	0.055	1.90 [0.70-5.18]	2.11E-01	I	0.055	0.032	0.036	1.84 [0.74-4.55]	1.89E-01	I	0.101	0.034	0.040	3.39 [1.98-5.81]	8.95E-06	I	0.082	0.036	0.041	2.68 [1.76-4.09]	4.21E-06
rs74915745	1	194609742	C/T	0.070	0.052	0.055	1.90 [0.70-5.18]	2.11E-01	I	0.055	0.032	0.036	1.84 [0.74-4.55]	1.89E-01	I	0.101	0.034	0.040	3.39 [1.98-5.81]	8.98E-06	I	0.082	0.036	0.041	2.68 [1.76-4.09]	4.22E-06
rs78251230	1	194610379	G/A	0.070	0.052	0.055	1.90 [0.70-5.18]	2.11E-01	I	0.055	0.032	0.036	1.84 [0.74-4.55]	1.89E-01	I	0.101	0.034	0.040	3.39 [1.98-5.81]	8.99E-06	I	0.082	0.036	0.041	2.68 [1.76-4.09]	4.23E-06
rs7015004	8	40337012	A/G	0.267	0.157	0.173	1.73 [0.95-3.14]	7.27E-02	I	0.210	0.128	0.142	1.97 [1.11-3.49]	2.03E-02	I	0.249	0.155	0.163	1.84 [1.31-2.57]	3.77E-04	I	0.243	0.151	0.161	1.84 [1.42-2.39]	4.23E-06
rs75644277	1	194612327	C/T	0.070	0.052	0.055	1.90 [0.70-5.18]	2.11E-01	I	0.055	0.032	0.036	1.84 [0.74-4.55]	1.89E-01	I	0.101	0.034	0.040	3.39 [1.98-5.80]	9.03E-06	I	0.082	0.036	0.041	2.68 [1.76-4.08]	4.24E-06
rs114119888	1	194641214	C/T	0.070	0.052	0.055	1.90 [0.70-5.18]	2.11E-01	I	0.055	0.032	0.036	1.84 [0.74-4.56]	1.87E-01	I	0.100	0.034	0.039	3.38 [1.97-5.80]	9.16E-06	I	0.082	0.036	0.041	2.68 [1.76-4.08]	4.24E-06
rs78106881	1	194641104	A/G	0.070	0.052	0.055	1.90 [0.70-5.18]	2.11E-01	I	0.055	0.032	0.036	1.84 [0.74-4.56]	1.87E-01	I	0.100	0.034	0.039	3.38 [1.97-5.80]	9.16E-06	I	0.082	0.036	0.041	2.68 [1.76-4.08]	4.24E-06
rs57932848	8	40337332	A/G	0.267	0.157	0.173	1.73 [0.95-3.14]	7.28E-02	I	0.210	0.128	0.142	1.97 [1.11-3.49]	2.03E-02	I	0.249	0.155	0.163	1.84 [1.31-2.57]	3.77E-04	I	0.242	0.151	0.161	1.84 [1.42-2.39]	4.24E-06
rs11581377	1	194614393	A/G	0.070	0.052	0.055	1.90 [0.70-5.18]	2.11E-01	I	0.055	0.032	0.036	1.84 [0.74-4.55]	1.89E-01	I	0.101	0.034	0.040	3.39 [1.98-5.80]	9.07E-06	I	0.082	0.036	0.041	2.68 [1.76-4.08]	4.25E-06
rs74500675	1	194615803	T/C	0.070	0.052	0.055	1.90 [0.70-5.18]	2.11E-01	I	0.055	0.032	0.036	1.84 [0.74-4.55]	1.89E-01	I	0.101	0.034	0.040	3.39 [1.98-5.80]	9.07E-06	I	0.082	0.036	0.041	2.68 [1.76-4.08]	4.25E-06
rs6983069	8	40337783	T/C	0.267	0.157	0.173	1.73 [0.95-3.14]	7.28E-02	I	0.210	0.128	0.142	1.97 [1.11-3.49]	2.03E-02	I	0.249	0.155	0.163	1.84 [1.31-2.57]	3.78E-04	I	0.242	0.151	0.161	1.84 [1.42-2.39]	4.25E-06
rs76504003	1	194613304	T/C	0.070	0.052	0.055	1.90 [0.70-5.18]	2.10E-01	I	0.055	0.032	0.036	1.83 [0.74-4.55]	1.90E-01	I	0.101	0.034	0.040	3.39 [1.98-5.80]	9.06E-06	I	0.082	0.036	0.041	2.68 [1.76-4.08]	4.26E-06
rs74910530	1	194615584	G/A	0.070	0.052	0.055	1.90 [0.70-5.18]	2.11E-01	I	0.055	0.032	0.036	1.84 [0.74-4.55]	1.89E-01	I	0.101	0.034	0.040	3.39 [1.98-5.80]	9.08E-06	I	0.082	0.036	0.041	2.68 [1.76-4.08]	4.26E-06
rs79189535	1	194616061	T/C	0.070	0.052	0.055	1.90 [0.70-5.18]	2.11E-01	I	0.055	0.032	0.036	1.84 [0.74-4.55]	1.89E-01	I	0.101	0.034	0.040	3.39 [1.98-5.80]	9.08E-06	I	0.082	0.036	0.041	2.68 [1.76-4.08]	4.26E-06
rs11583615	1	194616911	T/C	0.070	0.052	0.055	1.90 [0.70-5.18]	2.11E-01	I	0.055	0.032	0.036	1.84 [0.74-4.55]	1.89E-01	I	0.101	0.034	0.040	3.39 [1.98-5.80]	9.09E-06	I	0.082	0.036	0.041	2.68 [1.76-4.08]	4.26E-06
rs75888482	1	194642243	T/C	0.070	0.052	0.055	1.90 [0.70-5.18]	2.11E-01	I	0.055	0.032	0.036	1.84 [0.74-4.56]	1.87E-01	I	0.100	0.034	0.039	3.38 [1.97-5.80]	9.20E-06	I	0.082	0.036	0.041	2.68 [1.76-4.08]	4.26E-06
rs4557952	1	194635400	C/G	0.070	0.052	0.055	1.90 [0.70-5.18]	2.11E-01	I	0.055	0.032	0.036	1.84 [0.74-4.56]	1.87E-01	I	0.100	0.034	0.039	3.38 [1.97-5.78]	9.25E-06	I	0.082	0.036	0.041	2.68 [1.76-4.08]	4.26E-06
rs74689698	1	194637355	A/G	0.070	0.052	0.055	1.90 [0.70-5.18]	2.11E-01	I	0.055	0.032	0.036	1.84 [0.74-4.56]	1.87E-01	I	0.100	0.034	0.039	3.38 [1.97-5.79]	9.25E-06	I	0.082	0.036	0.041	2.68 [1.76-4.08]	4.27E-06
rs112989587	1	194619415	T/G	0.070	0.052	0.055	1.90 [0.70-5.18]	2.11E-01	I	0.055	0.032	0.036	1.84 [0.74-4.55]	1.89E-01	I	0.101	0.034	0.040	3.38 [1.97-5.80]	9.13E-06	I	0.082	0.036	0.041	2.68 [1.76-4.08]	4.28E-06
rs11582256	1	194621080	C/G	0.070	0.052	0.055	1.90 [0.70-5.18]	2.11E-01	I	0.055	0.032	0.036	1.84 [0.74-4.55]	1.89E-01	I	0.101	0.034	0.040	3.38 [1.97-5.80]	9.15E-06	I	0.082	0.036	0.041	2.68 [1.76-4.08]	4.28E-06
rs11582257	1	194621094	A/G	0.070	0.052	0.055	1.90 [0.70-5.18]	2.11E-01	I	0.055	0.032	0.036	1.84 [0.74-4.55]	1.89E-01	I	0.101	0.034	0.040	3.38 [1.97-5.80]	9.15E-06	I	0.082	0.036	0.041	2.68 [1.76-4.08]	4.28E-06
rs11586194	1	194621358	T/C	0.070	0.052	0.055	1.90 [0.70-5.18]	2.11E-01	I	0.055	0.032	0.036	1.84 [0.74-4.55]	1.89E-01	I	0.101	0.034	0.040	3.38 [1.97-5.80]	9.17E-06	I	0.082	0.036	0.041	2.68 [1.76-4.08]	4.29E-06
rs76503896	1	194622181	G/A	0.070	0.052	0.055	1.90 [0.70-5.18]	2.11E-01	I	0.055	0.032	0.036	1.84 [0.74-4.55]	1.89E-01	I	0.101	0.034	0.040	3.38 [1.97-5.80]	9.17E-06	I	0.082	0.036	0.041	2.68 [1.76-4.08]	4.29E-06

				GWAS1- subgroup A						GWAS1 - subgroup B						GWAS2						Meta-analysis				
SNP	Chr	Position	Risk/ other allel	RAF cases	RAF controls	RAF combined	RR [95% CI]	*P* value	Imputed	RAF cases	RAF controls	RAF combined	RR [95% CI]	*P* value	Imputed	RAF cases	RAF controls	RAF combined	RR [95% CI]	*P* value	Imputed	RAF cases	RAF controls	RAF combined	RR [95% CI]	*P* value
rs113074853	1	194621671	A/G	0.070	0.052	0.055	1.90 [0.70-5.18]	2.11E-01	I	0.055	0.032	0.036	1.84 [0.74-4.55]	1.89E-01	I	0.101	0.034	0.040	3.38 [1.97-5.80]	9.17E-06	I	0.082	0.036	0.041	2.68 [1.76-4.08]	4.29E-06
rs10958615	8	40340394	A/C	0.267	0.157	0.173	1.73 [0.95-3.14]	7.28E-02	I	0.210	0.128	0.142	1.97 [1.11-3.49]	2.03E-02	I	0.249	0.155	0.163	1.84 [1.31-2.57]	3.82E-04	I	0.242	0.151	0.161	1.84 [1.42-2.39]	4.30E-06
rs79472934	1	194628555	T/G	0.070	0.052	0.055	1.90 [0.70-5.18]	2.11E-01	I	0.055	0.032	0.036	1.84 [0.74-4.55]	1.89E-01	I	0.100	0.034	0.039	3.38 [1.97-5.78]	9.27E-06	I	0.082	0.036	0.041	2.68 [1.76-4.08]	4.32E-06
rs114931705	1	194647330	A/G	0.070	0.052	0.055	1.90 [0.70-5.18]	2.11E-01	I	0.055	0.032	0.036	1.84 [0.74-4.56]	1.88E-01	I	0.100	0.034	0.039	3.38 [1.97-5.79]	9.37E-06	I	0.082	0.036	0.041	2.68 [1.76-4.08]	4.32E-06
rs79641150	1	194650505	A/G	0.070	0.052	0.055	1.90 [0.70-5.18]	2.11E-01	I	0.055	0.032	0.036	1.84 [0.74-4.56]	1.88E-01	I	0.100	0.034	0.039	3.38 [1.97-5.79]	9.35E-06	I	0.082	0.036	0.041	2.68 [1.76-4.08]	4.33E-06
rs114717337	1	194631451	A/C	0.070	0.052	0.055	1.90 [0.70-5.18]	2.11E-01	I	0.055	0.032	0.036	1.84 [0.74-4.55]	1.89E-01	I	0.100	0.034	0.039	3.38 [1.97-5.78]	9.31E-06	I	0.082	0.036	0.041	2.68 [1.76-4.08]	4.34E-06
rs80018975	1	194631026	A/G	0.070	0.052	0.055	1.90 [0.70-5.18]	2.11E-01	I	0.055	0.032	0.036	1.84 [0.74-4.55]	1.89E-01	I	0.100	0.034	0.039	3.38 [1.97-5.78]	9.31E-06	I	0.082	0.036	0.041	2.68 [1.76-4.08]	4.34E-06
rs113209258	1	194624907	C/T	0.070	0.052	0.055	1.90 [0.70-5.18]	2.11E-01	I	0.055	0.032	0.036	1.84 [0.74-4.55]	1.89E-01	I	0.100	0.034	0.040	3.38 [1.97-5.79]	9.32E-06	I	0.082	0.036	0.041	2.68 [1.76-4.08]	4.34E-06
rs79294258	1	194624876	A/G	0.070	0.052	0.055	1.90 [0.70-5.18]	2.11E-01	I	0.055	0.032	0.036	1.84 [0.74-4.55]	1.89E-01	I	0.100	0.034	0.040	3.38 [1.97-5.79]	9.32E-06	I	0.082	0.036	0.041	2.68 [1.76-4.08]	4.34E-06
rs112607745	1	194624783	C/T	0.070	0.052	0.055	1.90 [0.70-5.18]	2.11E-01	I	0.055	0.032	0.036	1.84 [0.74-4.55]	1.89E-01	I	0.100	0.034	0.040	3.38 [1.97-5.79]	9.32E-06	I	0.082	0.036	0.041	2.68 [1.76-4.08]	4.34E-06
rs114068680	1	194645427	T/A	0.070	0.052	0.055	1.90 [0.70-5.18]	2.11E-01	I	0.055	0.032	0.036	1.84 [0.74-4.56]	1.88E-01	I	0.100	0.034	0.039	3.38 [1.97-5.79]	9.36E-06	I	0.082	0.036	0.041	2.68 [1.76-4.08]	4.34E-06
rs76090724	1	194644685	C/G	0.070	0.052	0.055	1.90 [0.70-5.18]	2.11E-01	I	0.055	0.032	0.036	1.84 [0.74-4.56]	1.88E-01	I	0.100	0.034	0.039	3.38 [1.97-5.79]	9.36E-06	I	0.082	0.036	0.041	2.68 [1.76-4.08]	4.34E-06
rs11577141	1	194643852	T/C	0.070	0.053	0.055	1.90 [0.70-5.18]	2.11E-01	I	0.055	0.032	0.036	1.84 [0.74-4.55]	1.88E-01	I	0.100	0.034	0.039	3.38 [1.97-5.79]	9.36E-06	I	0.082	0.036	0.041	2.68 [1.76-4.08]	4.34E-06
rs113830354	1	194636791	A/T	0.070	0.052	0.055	1.90 [0.70-5.18]	2.11E-01	I	0.055	0.032	0.036	1.84 [0.74-4.55]	1.89E-01	I	0.100	0.034	0.039	3.37 [1.97-5.78]	9.37E-06	I	0.082	0.036	0.041	2.68 [1.76-4.08]	4.36E-06
rs112826357	1	194635178	T/C	0.070	0.052	0.055	1.90 [0.70-5.18]	2.11E-01	I	0.055	0.032	0.036	1.84 [0.74-4.55]	1.89E-01	I	0.100	0.034	0.039	3.37 [1.97-5.78]	9.38E-06	I	0.082	0.036	0.041	2.68 [1.76-4.07]	4.36E-06
rs74921449	1	194636225	A/G	0.070	0.052	0.055	1.90 [0.70-5.18]	2.11E-01	I	0.055	0.032	0.036	1.84 [0.74-4.55]	1.89E-01	I	0.100	0.034	0.039	3.37 [1.97-5.78]	9.38E-06	I	0.082	0.036	0.041	2.68 [1.76-4.07]	4.36E-06
rs115650236	4	59668829	G/A													0.076	0.027	0.031	5.11 [2.54-10.26]	*4.51E-06*	I	0.053	0.026	0.029	5.11 [2.54-10.26]	*4.51E-06*
rs72765668	5	50958023	G/A	0.191	0.098	0.111	2.50 [1.21-5.17]	*1.31E-02*	I	0.130	0.098	0.103	*1.52 [0.74-3.10]*	*2.55E-01*	I	0.175	0.098	0.105	2.24 [1.48-3.38]	*1.27E-04*	I	0.167	0.098	0.105	2.12 [1.54-2.92]	*4.52E-06*
rs7824285	8	40285421	T/C	0.267	0.161	0.176	1.70 [0.94-3.09]	8.18E-02	I	0.209	0.131	0.144	1.90 [1.07-3.35]	2.75E-02	-	0.259	0.162	0.170	1.84 [1.32-2.55]	3.05E-04	-	0.248	0.157	0.167	1.82 [1.41-2.36]	4.95E-06
rs7689350	4	76207570	C/A	0.228	0.109	0.126	2.54 [1.32-4.90]	5.33E-03	I	0.155	0.096	0.106	1.66 [0.91-3.05]	1.00E-01	-	0.197	0.120	0.127	1.86 [1.30-2.68]	7.61E-04	I	0.192	0.115	0.123	1.93 [1.45-2.55]	4.95E-06
rs62505233	8	40299662	T/A	0.265	0.159	0.174	1.70 [0.93-3.10]	8.26E-02	I	0.208	0.128	0.142	1.94 [1.10-3.44]	2.31E-02	I	0.254	0.159	0.167	1.83 [1.31-2.56]	3.63E-04	I	0.244	0.154	0.164	1.83 [1.41-2.37]	5.13E-06
rs17629112	8	40333751	T/C	0.267	0.157	0.173	1.73 [0.95-3.14]	7.24E-02	I	0.209	0.127	0.141	1.96 [1.11-3.46]	2.10E-02	-	0.249	0.156	0.164	1.82 [1.30-2.55]	4.57E-04	I	0.242	0.152	0.162	1.83 [1.41-2.38]	5.23E-06
rs78508457	1	194616426	T/C	0.071	0.056	0.058	1.71 [0.63-4.62]	2.91E-01	I	0.055	0.032	0.036	1.84 [0.74-4.55]	1.89E-01	I	0.102	0.034	0.040	3.42 [2.00-5.86]	7.18E-06	I	0.083	0.037	0.042	2.65 [1.74-4.03]	5.26E-06
rs62506987	8	40333463	A/C	0.267	0.157	0.173	1.73 [0.95-3.14]	7.23E-02	I	0.209	0.127	0.141	1.96 [1.11-3.47]	2.09E-02	I	0.249	0.156	0.164	1.82 [1.30-2.55]	4.62E-04	I	0.242	0.152	0.162	1.83 [1.41-2.38]	5.26E-06
rs192356617	1	194638769	C/A	0.080	0.050	0.054	2.73 [0.95-7.87]	6.21E-02	I	0.051	0.031	0.034	1.88 [0.72-4.89]	1.97E-01	I	0.091	0.033	0.038	3.34 [1.87-5.96]	*4.68E-05*	I	0.078	0.035	0.04	2.84 [1.81-4.45]	*5.37E-06*
rs62505258	8	40310698	C/G	0.267	0.160	0.175	1.71 [0.94-3.10]	7.96E-02	I	0.209	0.128	0.142	1.95 [1.10-3.45]	2.17E-02	I	0.254	0.160	0.168	1.82 [1.30-2.53]	4.20E-04	I	0.245	0.155	0.165	1.82 [1.41-2.36]	5.43E-06
rs72748303	1	208973633	A/G	0.046	0.027	0.029	3.15 [0.85-11.65]	8.54E-02	I	0.072	0.016	0.026	5.39 [1.85-15.75]	2.05E-03	I	0.058	0.024	0.027	2.81 [1.44-5.46]	2.41E-03	I	0.059	0.023	0.027	3.33 [1.98-5.60]	5.59E-06
rs72746301	1	208973521	A/T	0.046	0.027	0.030	3.11 [0.84-11.47]	8.92E-02	I	0.072	0.016	0.026	5.36 [1.84-15.64]	2.10E-03	I	0.058	0.024	0.027	2.8 [1.44-5.46]	2.37E-03	I	0.059	0.023	0.027	3.32 [1.98-5.57]	5.79E-06
rs72746298	1	208973461	A/G	0.046	0.027	0.030	3.09 [0.84-11.40]	9.03E-02	I	0.072	0.016	0.026	5.35 [1.83-15.61]	2.15E-03	I	0.058	0.024	0.027	2.81 [1.44-5.46]	2.36E-03	I	0.059	0.023	0.027	3.31 [1.97-5.57]	5.93E-06
rs3827410	22	46879687	C/A	0.395	0.353	0.641	1.33 [0.79-2.23]	2.84E-01	-	0.512	0.371	0.605	1.93 [1.24-3.00]	3.60E-03	I	0.460	0.346	0.644	1.67 [1.25-2.24]	*5.36E-04*	I	0.46	0.351	0.363	1.66 [1.33-2.07]	*6.13E-06*
rs7775008	6	68699843	C/A	0.442	0.387	0.394	1.3 [0.79-2.12]	2.97E-01	I	0.564	0.380	0.411	1.91 [1.26-2.91]	2.35E-03	-	0.505	0.382	0.392	1.63 [1.23-2.17]	7.08E-04	-	0.507	0.382	0.396	1.63 [1.32-2.01]	6.21E-06
rs1222043	1	96625497	C/G	0.044	0.036	0.037	2.53 [0.64-10.09]	1.87E-01	I							0.094	0.035	0.040	3.59 [2.02-6.38]	*1.39E-05*	I	0.064	0.036	0.039	3.41 [2.00-5.80]	*6.24E-06*
rs113641325	12	79539759	C/G	0.220	0.124	0.138	2.62 [1.27-5.42]	*9.44E-03*	I	0.214	0.140	0.153	1.62 [0.92-2.83]	*9.40E-02*	I	0.195	0.123	0.129	2.02 [1.35-3.01]	*5.68E-04*	I	0.206	0.126	0.135	1.98 [1.47-2.67]	*6.49E-06*
rs12345625	9	31479005	T/C	0.093	0.029	0.038	4.69 [1.67-13.21]	3.40E-03	-	0.072	0.034	0.040	2.24 [0.94-5.35]	7.01E-02	I	0.068	0.030	0.033	2.5 [1.41-4.44]	1.75E-03	I	0.074	0.03	0.035	2.72 [1.76-4.20]	6.59E-06
rs62365476	5	50803801	A/G	0.453	0.332	0.350	1.97 [1.12-3.47]	*1.83E-02*	I	0.319	0.284	0.290	1.19 [0.76-1.85]	4.47E-01	I	0.447	0.308	0.320	1.82 [1.37-2.42]	3.58E-05	I	0.414	0.308	0.32	1.66 [1.33-2.07]	*6.65E-06*
rs73077895	7	28819842	A/G													0.056	0.013	0.017	5.35 [2.58-11.11]	6.84E-06	I	0.033	0.016	0.017	5.35 [2.58-11.11]	6.84E-06
rs56189125	7	28855348	G/A													0.053	0.013	0.016	5.98 [2.74-13.03]	6.87E-06	I	0.033	0.015	0.017	5.98 [2.74-13.03]	6.87E-06
rs2299118	7	28819119	G/C													0.056	0.013	0.017	5.35 [2.57-11.10]	6.91E-06	I	0.033	0.016	0.017	5.35 [2.57-11.10]	6.91E-06
rs1877775	6	68673028	G/T	0.419	0.359	0.368	1.34 [0.81-2.19]	2.51E-01	I	0.555	0.346	0.382	2.08 [1.38-3.15]	4.98E-04	-	0.464	0.356	0.365	1.53 [1.16-2.03]	2.83E-03	-	0.478	0.355	0.368	1.62 [1.31-2.00]	6.97E-06
rs76239813	11	124909582	A/T	0.071	0.017	0.025	10.01 [2.66-37.62]	*6.48E-04*	I	0.061	0.015	0.022	5.91 [1.84-19.00]	2.85E-03	I							0.039	0.016	0.018	*7.44 [3.10-17.87]*	*7.00E-06*
rs10976332	9	7503337	A/G	0.215	0.161	0.168	1.63 [0.84-3.15]	1.51E-01	I	0.297	0.208	0.224	1.74 [1.06-2.84]	2.79E-02	I	0.317	0.202	0.212	1.81 [1.32-2.48]	2.55E-04	I	0.291	0.197	0.207	1.76 [1.38-2.26]	7.02E-06
rs72746296	1	208973133	G/A	0.046	0.027	0.030	2.96 [0.81-10.90]	1.02E-01	I	0.072	0.016	0.026	5.25 [1.81-15.21]	2.27E-03	I	0.058	0.024	0.027	2.79 [1.44-5.41]	2.43E-03	I	0.06	0.024	0.028	3.27 [1.95-5.47]	7.02E-06
rs76754339	6	118104136	G/A													0.055	0.017	0.020	5.7 [2.67-12.18]	7.05E-06	I	0.034	0.016	0.018	5.70 [2.67-12.18]	7.05E-06
rs6861649	5	50864788	C/T	0.733	0.657	0.668	1.49 [0.85-2.62]	1.63E-01	I	0.650	0.624	0.629	1.11 [0.73-1.71]	6.21E-01	I	0.806	0.621	0.636	2.49 [1.76-3.53]	2.75E-07	I	0.75	0.627	0.64	1.75 [1.37-2.23]	7.35E-06
rs72746294	1	208972947	A/G	0.046	0.027	0.030	2.90 [0.79-10.63]	1.08E-01	I	0.072	0.016	0.026	5.19 [1.79-15.00]	2.39E-03	I	0.058	0.024	0.027	2.78 [1.43-5.39]	2.45E-03	I	0.06	0.024	0.028	3.24 [1.94-5.43]	7.77E-06
rs2400369	12	79496494	A/G	0.232	0.124	0.139	2.84 [1.42-5.70]	3.30E-03	I	0.206	0.147	0.157	1.37 [0.81-2.33]	2.45E-01	I	0.203	0.120	0.127	1.94 [1.35-2.80]	3.58E-04	I	0.21	0.125	0.134	1.88 [1.42-2.48]	7.80E-06
rs12178826	6	68632721	G/T	0.409	0.358	0.365	1.35 [0.82-2.22]	2.36E-01	I	0.566	0.349	0.386	2.17 [1.43-3.29]	2.97E-04	I	0.457	0.354	0.363	1.5 [1.13-1.99]	4.46E-03	I	0.476	0.354	0.367	1.62 [1.31-1.99]	8.03E-06
rs4631671	1	194633947	T/C	0.047	0.046	0.046	1.45 [0.44-4.84]	5.42E-01	I	0.039	0.028	0.029	1.54 [0.52-4.58]	4.34E-01	I	0.099	0.031	0.037	3.79 [2.18-6.59]	2.23E-06	I	0.072	0.033	0.037	2.82 [1.79-4.45]	8.06E-06
rs1514921	12	79481756	G/A	0.232	0.124	0.139	2.84 [1.42-5.71]	3.27E-03	I	0.206	0.147	0.157	1.37 [0.81-2.33]	2.45E-01	I	0.203	0.120	0.127	1.94 [1.35-2.80]	3.77E-04	I	0.21	0.125	0.134	1.88 [1.42-2.47]	8.15E-06
rs12547312	8	40333291	A/G	0.267	0.158	0.174	1.72 [0.95-3.12]	7.55E-02	-	0.209	0.127	0.141	1.96 [1.11-3.47]	2.08E-02	I	0.250	0.159	0.167	1.78 [1.28-2.48]	6.91E-04	I	0.243	0.154	0.164	1.8 [1.39-2.34]	8.15E-06
rs6912774	6	68609707	G/A	0.395	0.361	0.366	1.28 [0.78-2.10]	3.31E-01	I	0.573	0.350	0.388	2.17 [1.44-3.28]	2.24E-04	-	0.459	0.355	0.364	1.51 [1.14-2.00]	3.81E-03	-	0.476	0.355	0.368	1.61 [1.31-1.99]	8.26E-06
rs12198681	6	68633976	G/T	0.414	0.361	0.369	1.36 [0.83-2.23]	2.26E-01	I	0.572	0.351	0.389	2.17 [1.44-3.29]	2.36E-04	I	0.455	0.355	0.364	1.48 [1.12-1.96]	5.57E-03	-	0.477	0.355	0.369	1.61 [1.31-1.99]	8.27E-06
rs10068374	5	50815539	G/C	0.452	0.333	0.350	1.96 [1.12-3.45]	*1.92E-02*	I	0.319	0.284	0.290	1.19 [0.76-1.85]	4.48E-01	I	0.445	0.309	0.320	1.8 [1.36-2.40]	4.48E-05	I	0.413	0.308	0.32	1.65 [1.32-2.06]	*8.28E-06*
rs6858981	5	50826507	T/C	0.452	0.331	0.348	1.97 [1.12-3.46]	*1.86E-02*	I	0.319	0.284	0.290	1.19 [0.76-1.86]	4.42E-01	I	0.443	0.307	0.318	1.8 [1.36-2.39]	4.76E-05	I	0.412	0.307	0.318	1.65 [1.32-2.06]	*8.29E-06*
rs75518401	12	79533690	A/G	0.232	0.149	0.161	2.15 [1.08-4.30]	*2.98E-02*	I	0.269	0.167	0.184	1.88 [1.12-3.16]	*1.76E-02*	I	0.222	0.149	0.155	1.81 [1.24-2.62]	*1.84E-03*	I	0.236	0.151	0.161	1.88 [1.42-2.48]	*8.32E-06*
rs28886695	4	76183741	G/T	0.228	0.106	0.124	2.68 [1.37-5.23]	3.84E-03	I	0.154	0.094	0.104	1.69 [0.92-3.11]	8.91E-02	I	0.187	0.116	0.122	1.8 [1.25-2.60]	1.70E-03	I	0.187	0.111	0.119	1.91 [1.44-2.54]	8.35E-06
rs11587150	1	194643906	C/T	0.073	0.056	0.058	1.89 [0.69-5.16]	2.15E-01	I	0.067	0.037	0.042	2.04 [0.86-4.83]	1.06E-01	I	0.106	0.040	0.046	2.94 [1.75-4.94]	4.68E-05	I	0.088	0.042	0.047	2.52 [1.68-3.79]	8.39E-06
rs72746213	1	208922863	T/A	0.090	0.044	0.050	3.31 [1.16-9.43]	2.49E-02	I	0.094	0.046	0.054	2.19 [0.98-4.90]	5.67E-02	I	0.093	0.041	0.046	2.53 [1.48-4.32]	6.73E-04	I	0.092	0.042	0.048	2.54 [1.69-3.83]	8.40E-06
rs181099388	4	39462030	G/T	0.067	0.022	0.028	4.12 [1.22-13.89]	2.23E-02	I	0.040	0.025	0.028	2.27 [0.69-7.51]	1.80E-01	I	0.082	0.030	0.034	2.76 [1.60-4.77]	2.61E-04	I	0.068	0.028	0.032	2.84 [1.80-4.50]	8.41E-06
rs184483498	1	170906758	A/G							0.110	0.069	0.076	1.87 [0.83-4.21]	*1.31E-01*	I	0.146	0.071	0.077	2.8 [1.75-4.49]	*1.92E-05*	I	0.132	0.071	0.078	2.53 [1.68-3.80]	*8.43E-06*
rs7714549	5	50832849	C/A	0.453	0.333	0.350	1.96 [1.12-3.45]	*1.91E-02*	I	0.321	0.284	0.290	1.2 [0.77-1.87]	4.24E-01	I	0.443	0.308	0.319	1.8 [1.35-2.39]	5.16E-05	I	0.413	0.308	0.319	1.65 [1.32-2.06]	*8.45E-06*
rs12648574	4	76185683	C/T	0.228	0.106	0.124	2.68 [1.37-5.23]	3.85E-03	I	0.154	0.094	0.104	1.69 [0.92-3.11]	8.91E-02	I	0.187	0.116	0.122	1.8 [1.25-2.59]	1.72E-03	I	0.187	0.111	0.119	1.91 [1.44-2.53]	8.46E-06
rs2048702	6	68638309	T/C	0.414	0.361	0.369	1.36 [0.83-2.23]	2.26E-01	I	0.571	0.351	0.389	2.17 [1.44-3.29]	2.41E-04	I	0.455	0.355	0.364	1.48 [1.12-1.96]	5.61E-03	I	0.477	0.356	0.369	1.61 [1.31-1.99]	8.47E-06
rs28792753	4	76183768	T/C	0.228	0.106	0.124	2.68 [1.37-5.23]	3.84E-03	I	0.154	0.094	0.104	1.69 [0.92-3.11]	8.91E-02	I	0.187	0.116	0.122	1.8 [1.25-2.59]	1.73E-03	I	0.187	0.111	0.12	1.91 [1.44-2.53]	8.51E-06
rs55654093	8	40319687	A/T	0.266	0.156	0.171	1.73 [0.95-3.16]	7.22E-02	I	0.206	0.124	0.138	2.01 [1.13-3.60]	1.81E-02	I	0.247	0.159	0.166	1.78 [1.27-2.49]	8.38E-04	I	0.24	0.153	0.162	1.81 [1.40-2.36]	8.55E-06
rs2137885	6	68629446	T/C	0.413	0.358	0.366	1.37 [0.84-2.26]	2.09E-01	I	0.565	0.349	0.386	2.15 [1.42-3.25]	3.14E-04	I	0.454	0.354	0.362	1.49 [1.13-1.97]	5.31E-03	I	0.475	0.354	0.367	1.61 [1.31-1.99]	8.61E-06
rs56376152	7	28855309	A/G													0.052	0.012	0.016	5.87 [2.69-12.80]	8.66E-06	I	0.031	0.015	0.016	5.87 [2.69-12.80]	8.66E-06
rs180692175	2	133631058	G/T	0.049	0.021	0.025	3.97 [0.79-20.02]	*9.53E-02*	I	0.053	0.025	0.030	3.94 [1.08-14.36]	*3.76E-02*	I	0.058	0.024	0.027	*3.94 [1.85-8.38]*	*3.71E-04*	I	0.055	0.024	0.027	3.94 [2.15-7.22]	*8.68E-06*
rs66507483	4	76187901	T/C	0.228	0.106	0.124	2.68 [1.37-5.23]	3.87E-03	I	0.154	0.094	0.104	1.69 [0.92-3.11]	8.90E-02	I	0.187	0.116	0.122	1.8 [1.24-2.59]	1.78E-03	I	0.187	0.111	0.12	1.91 [1.43-2.53]	8.76E-06
rs55786015	4	76204192	T/C	0.227	0.105	0.123	2.69 [1.38-5.26]	3.83E-03	I	0.154	0.094	0.104	1.69 [0.92-3.11]	8.87E-02	I	0.187	0.116	0.123	1.79 [1.24-2.59]	1.79E-03	I	0.187	0.111	0.119	1.91 [1.43-2.53]	8.80E-06
rs6919995	6	68630430	A/G	0.411	0.357	0.365	1.37 [0.83-2.26]	2.19E-01	I	0.559	0.348	0.384	2.13 [1.40-3.23]	*3.88E-04*	I	0.453	0.352	0.360	1.5 [1.13-1.98]	4.66E-03	I	0.472	0.352	0.365	1.61 [1.31-1.99]	*8.89E-06*
rs6894368	5	50825829	T/C	0.452	0.332	0.349	1.96 [1.12-3.44]	*1.92E-02*	I	0.320	0.284	0.290	1.19 [0.76-1.86]	4.43E-01	I	0.445	0.309	0.320	1.8 [1.35-2.38]	5.06E-05	I	0.413	0.308	0.32	1.65 [1.32-2.05]	*8.98E-06*
rs72748307	1	208974152	C/T	0.036	0.025	0.027	2.67 [0.64-11.17]	1.80E-01	I	0.067	0.016	0.024	5.22 [1.75-15.64]	3.12E-03	I	0.057	0.023	0.026	2.96 [1.51-5.78]	1.55E-03	I	0.055	0.022	0.026	3.33 [1.96-5.67]	9.10E-06
rs7310309	12	79811429	T/C	0.285	0.144	0.836	2.52 [1.36-4.65]	*3.27E-03*	I	0.227	0.162	0.827	1.42 [0.83-2.41]	1.97E-01	I	0.231	0.149	0.844	1.84 [1.29-2.63]	7.91E-04	I	0.241	0.15	0.16	1.83 [1.40-2.39]	*9.26E-06*
rs72748306	1	208974054	C/T	0.036	0.025	0.027	2.63 [0.63-11.02]	1.87E-01	I	0.067	0.016	0.024	5.21 [1.74-15.58]	3.16E-03	I	0.058	0.023	0.026	2.96 [1.51-5.78]	1.53E-03	I	0.056	0.022	0.026	3.32 [1.95-5.65]	9.39E-06
rs74543572	1	208974031	A/C	0.036	0.025	0.027	2.62 [0.62-10.98]	1.89E-01	I	0.067	0.016	0.024	5.20 [1.74-15.57]	3.17E-03	I	0.058	0.023	0.026	2.96 [1.51-5.78]	1.52E-03	I	0.056	0.022	0.026	3.32 [1.95-5.65]	9.44E-06

Supplementary Table 2. Meta-anaylsis results in classic bladder exstrophy

				GWAS1- subgroup A						GWAS1 - subgroup B						GWAS2						Meta-analysis				
SNP	Chr	Position	Risk/ other allel	RAF cases	RAF controls	RAF combined	RR [95% CI]	*P* value	Imputed	RAF cases	RAF controls	RAF combined	RR [95% CI]	*P* value	Imputed	RAF cases	RAF controls	RAF combined	RR [95% CI]	*P* value	Imputed	RAF cases	RAF controls	RAF combined	RR [95% CI]	*P* value
rs2950386	12	79484134	G/A	0.233	0.125	0.141	2.78 [1.39-5.55]	3.73E-03	-	0.209	0.148	0.158	1.39 [0.82-2.35]	2.22E-01	-	0.205	0.122	0.129	1.91 [1.33-2.75]	4.75E-04	-	0.212	0.127	0.136	1.86 [1.41-2.45]	9.49E-06
rs62000819	14	24512897	G/C							0.556	0.480	0.493	1.58 [1.00-2.49]	*5.08E-02*	I	0.630	0.491	0.503	*1.92 [1.40-2.64]*	*5.47E-05*	I	0.584	0.493	0.503	*1.80 [1.39-2.34]*	*9.52E-06*
rs115246907	1	208973973	A/C	0.036	0.025	0.027	2.60 [0.62-10.90]	1.92E-01	I	0.067	0.016	0.024	5.19 [1.74-15.53]	3.20E-03	I	0.058	0.023	0.026	2.96 [1.51-5.78]	1.51E-03	I	0.056	0.022	0.026	3.32 [1.95-5.64]	9.63E-06
rs72660783	5	108627280	T/C	0.025	0.041	0.039	0.21 [0.02-2.69]	*2.32E-01*	I							0.090	0.037	0.041	*5.87 [2.90-11.87]*	*8.48E-07*	I	0.06	0.038	0.04	*4.63 [2.35-9.12]*	*9.65E-06*
rs7834492	8	40319688	A/T	0.266	0.156	0.171	1.73 [0.95-3.16]	7.23E-02	I	0.206	0.125	0.139	2.01 [1.13-3.59]	1.83E-02	I	0.247	0.159	0.167	1.76 [1.26-2.47]	9.47E-04	I	0.24	0.153	0.163	1.81 [1.39-2.35]	9.75E-06
rs72748309	1	208974561	A/G	0.035	0.023	0.025	2.91 [0.70-12.13]	1.42E-01	I	0.064	0.015	0.023	5.16 [1.71-15.54]	3.55E-03	I	0.054	0.022	0.024	2.95 [1.49-5.84]	1.92E-03	I	0.053	0.021	0.024	3.36 [1.96-5.76]	9.94E-06
