# Supplementary material for: Genome-wide Association Study and Meta-Analysis Identify ISL1 as Genome-wide Significant Susceptibility Gene for Bladder Exstrophy
Source: PLoS Genet. 2015 Mar 12;11(3):e1005024. doi: 10.1371/journal.pgen.1005024 (PMC4357422; doi:10.1371/journal.pgen.1005024)
Supplement: S3 Table — F, forward; R, reverse. (PDF) [file pgen.1005024.s003.pdf]

Supplementary Table 3. Primers (5'->3' direction) used for ISL1 sequence analysis

ISL1-1F	GATAATCAGAACAGCTGCGCC
ISL1-1R	TCCAACTCCAAAGAGCCCTTG
ISL1-2F	AAACCTCCCAGAGTACGCCC
ISL1-2R	GTGGGGAGATTCAGGGAAATC
ISL1-3F	GATCTTGGGCCAGGGAAGTG
ISL1-3R	GCAGGCAAACACTACGACCAC
ISL1-4F	TGTCCTGAGTATCTCGGGCG
ISL1-4R	CGATCCTGCGTACCAGGAAC
ISL1-5F	AGGTACGGCGGATTAACTGAGT
ISL1-5R	GGTTTCTCCCCAACCCTGAG
ISL1-6F	TGGGAAAGTGAGAGGATTTCTTC
ISL1-6R	TGATTCAGTTTTCATTGACTGGG
